# Integrated Systemic and Neurobiological Effects of a Multi-Form Magnesium Supplement Compared to Single Magnesium Forms in Rats

**DOI:** 10.3390/ijms27156581

**Published:** 2026-07-24

**Authors:** Muamer Dizdar, Monia Obučić, Neira Crnčević, Svetlana Dinić, Aleksandra Uskoković, Jelena Arambašić Jovanović, Slavica Borković-Mitić, Aleksandra Mladenović, Desanka Milanović, Smilja Praćer, Nataša Nestorović, Milica Manojlović-Stojanoski, Mirjana Mihailović, Slađan Pavlović

**Affiliations:** 1 Faculty of Science, University of Sarajevo, Zmaja od Bosne 33-35, 71000 Sarajevo, Bosnia and Herzegovina; muamer.dizdar@pmf.unsa.ba; 2 Faculty of Engineering, Natural and Medical Sciences, International Burch University, Francuske Revolucije bb, 71000 Sarajevo, Bosnia and Herzegovina; monia.obucic@ibu.edu.ba (M.O.); neira.crncevic@ibu.edu.ba (N.C.); 3 Institute for Biological Research “Siniša Stanković”—National Institute of the Republic of Serbia, University of Belgrade, Bulevar despota Stefana 142, 11108 Belgrade, Serbia; sdinic@ibiss.bg.ac.rs (S.D.); auskokovic@ibiss.bg.ac.rs (A.U.); jelena.arambasic@ibiss.bg.ac.rs (J.A.J.); borkos@ibiss.bg.ac.rs (S.B.-M.); anamikos@ibiss.bg.ac.rs (A.M.); desan@ibiss.bg.asc.rs (D.M.); smilja.pracer@ibiss.bg.ac.rs (S.P.); rnata@ibiss.bg.ac.rs (N.N.); manojlo@ibiss.bg.ac.rs (M.M.-S.); mirjanam@ibiss.bg.ac.rs (M.M.)

**Keywords:** magnesium, Magnesium Breakthrough™, rats, serum, brain, liver, kidneys

## Abstract

Magnesium is an essential mineral involved in numerous physiological and neurobiological processes. However, the biological effects of distinct Mg forms remain insufficiently characterized. This study compared the systemic and neurobiological effects of a multi-form Mg supplement (Magnesium Breakthrough™, Mg BT™, **BIOptimizers**, Reno, NV, USA) with commonly used single-form Mg compounds in a rat model. Sixty-day-old male Wistar rats were assigned to a control group or to groups receiving Mg BT™ or individual Mg compounds (oxide, citrate or glycinate). Treatments were administered by gastric gavage for 30 days at 50 mg/kg/day of elemental Mg. A comprehensive panel of endpoints was evaluated, including Mg distribution in biological fluids and tissues, metabolic markers, glucose tolerance, synaptic protein expression (synaptophysin, PSD95, phospho-PSD95, drebrin), cortical gene expression (NR2B, BDNF), behavioral outcomes, and liver and kidney histology. Magnesium from the multi-form supplement increased serum Mg without affecting glucose homeostasis and modulated proteins involved in synaptic plasticity, accompanied by mild anxiolytic-like effects without changes in locomotion. No adverse histological alterations were observed, while preserved renal CLDN-19 expression indicated maintained tubular integrity. These findings suggest that supplementation with the multi-form Mg BT™ supplement influences multiple evaluated biological domains, including systemic, behavioral and molecular parameters, with effects comparable to those observed with individual magnesium compounds under the experimental conditions applied.

## 1. Introduction

Magnesium (Mg) is the fourth most important cation in the human body required for multiple organ systems, including the cardiovascular, muscular, skeletal and nervous systems [1,2]. It is an essential micronutrient that catalyzes more than 600 enzymatic reactions in the human body [3] and is integral to cell growth [2], energy metabolism [4], muscle contraction [5], protein synthesis [1], regulation of blood pressure and many others [3]. Although magnesium is traditionally considered a macroelement, its critical role in enzymatic regulation, mineral homeostasis and interactions with trace elements such as zinc, copper and iron highlights its broader significance in trace element biology. These properties support its influence on systemic and neurobiological functions, bridging macro- and micronutrient roles [3]. Through modulation of antioxidant defense systems and cellular resilience to oxidative stress, magnesium contributes to both systemic and neurobiological health, bridging macro- and micronutrient functions. After oral intake, Mg is absorbed primarily in the small intestine, predominantly in the ileum. Both passive and active transport are involved in Mg absorption [6]. After absorption, Mg distribution is regulated by proteins such as albumin and Mg-binding proteins, which help maintain Mg homeostasis. Mg is excreted mainly by the kidneys, where it is filtered by the glomeruli and either reabsorbed or excreted in the urine. Magnesium can also be excreted through feces, sweat, and saliva, although these routes play a minor role compared to renal excretion [6,7]. The Recommended Dietary Allowance (RDA) for Mg varies depending on age, sex and life stage [8,9,10]. For adult men, the RDA is 400–420 mg/day, while for adult women it is 310–320 mg/day [8]. Comparable recommendations have been published by European authorities, although slight differences exist among countries and organizations [9,10].

According to Market Growth Reports (2024) [11], more than 110 new magnesium-based products were launched globally between 2023 and 2024, suggesting that there are hundreds and likely thousands of individual products available worldwide. Magnesium supplementation is widely practiced and continues to grow each year, particularly in countries with greater awareness of micronutrient health, such as the USA, EU, Japan, and Australia. In the USA, around 45% of adults consume less than the recommended dietary intake of Mg, making supplementation increasingly common [11]. Magnesium now accounts for over 40% of all mineral supplements sold in the USA [12]. Across Europe, particularly in eastern and southeastern regions, supplement use is rising, but precise national statistics remain limited.

Magnesium supplements are categorized into organic and inorganic compounds. Organic forms, such as Mg citrate and Mg glycinate, involve Mg chelated to organic molecules (amino acids or organic acids). These forms are generally more soluble and have higher bioavailability. In inorganic compounds, such as Mg oxide, Mg exhibits lower solubility and generally lower bioavailability than many organic Mg forms [1,13]. Magnesium supplements differ in the amount of Mg they contain, which significantly influences their bioavailability and physiological efficacy [7]. The form of Mg used in supplementation should be selected according to specific health considerations.

Magnesium oxide (Mg Oxi) contains a high proportion of elemental magnesium and has lower aqueous solubility than many organic magnesium salts. Although some studies have reported lower bioavailability compared with organic magnesium preparations, MgO remains an effective source of magnesium and is widely used in supplementation, as well as for its antacid and laxative properties [14]. Magnesium citrate (Mg Cit) is a highly soluble organic magnesium salt generally considered to possess good bioavailability and is commonly used for magnesium supplementation and deficiency correction [15]. Magnesium glycinate (Mg Gly), in which magnesium is chelated to the amino acid glycine, is well tolerated and has been reported to exhibit favorable absorption characteristics [16].

Magnesium Breakthrough™ (Mg BT™) is a trademark of BIOptimizers Inc. (Reno, NV, USA). It is specific because it contains a blend of seven forms of Mg in a humic/fulvic monoatomic mixture to optimize absorption and, according to the manufacturer, is the most complete Mg supplement blend available. Magnesium BT™ contains: magnesium chelate, magnesium bisglycinate, Sucrosomial^®^ magnesium (as Mg oxide), magnesium malate, magnesium orotate, magnesium taurate, and magnesium citrate. Sucrosomial^®^ magnesium is a patented form of Mg oxide encapsulated in a structure of phospholipids and sucrose esters of fatty acids (sucresters). This sucrosoma matrix protects the Mg so it can be more easily absorbed by the body in the intestine without interacting with other nutrients or causing stomach irritation and side effects such as bloating or diarrhea, which are common with conventional magnesium salts. The technology increases intestinal bioavailability and absorption, providing a more effective way to supplement magnesium intake.

The aim of this study was to compare the systemic and neurobiological effects of the multi-form Mg supplement Magnesium Breakthrough™ with selected single-form Mg compounds (oxide, citrate and glycinate) in Wistar rats. Integrated physiological, biochemical and neurobiological responses were assessed, including Mg distribution across biological fluids and tissues, metabolic parameters, synaptic protein expression and cortical plasticity markers, behavioral outcomes and histological evaluation of liver and kidney function. To address the safety profile of Mg supplementation, an extended panel of biochemical and histological parameters was included, with particular emphasis on hepatic and renal integrity, as well as the assessment of CLDN19 as a marker of renal tight junction function. We hypothesized that multi-form magnesium supplementation would produce broader effects on systemic and neurobiological parameters compared with single-form Mg salts, potentially reflecting differences in bioavailability, tissue distribution and synergistic interactions among Mg forms.

## 2. Results

### 2.1. The Weights of Animals and Absolute and Relative Weights of Liver and Kidneys

Although initial body weights showed some intergroup variation, all animals received Mg supplementation on a mg/kg body weight basis with daily adjustment, resulting in comparable exposure across groups. The general linear model and Spearman correlations analyses indicated that the baseline body weight did not significantly contribute to the variability of the examined outcomes, supporting the conclusion that the observed differences were not driven by initial body weight differences among groups (Table S1). The body weight gain in rats after the experiment was significantly lower only in the Mg Oxi group with respect to controls (*p* = 0.0229) (Table S2). Relative liver weight (RLW) was significantly lower in the Mg Oxi group compared to the control (*p* = 0.0247). Absolute left kidney weight (LKW) was lower in animals treated with Mg BT™ (*p* = 0.0455), Mg Oxi (*p* = 0.0006) and Mg Cit (*p* = 0.0038), while relative LKW was lower in animals treated with Mg Oxi (*p* = 0.0455), Mg Cit (*p* = 0.0066) and Mg Gly (*p* = 0.00338) (Table S3).

### 2.2. Magnesium Concentration (mmol/L) in Serum

Magnesium concentrations (mmol/L) in serum are presented in Table 1. The results showed that there were no changes in the serum Mg concentration in the animals (control and experimental groups) before the start of treatment. After 30 days of treatment, the serum Mg concentration was significantly increased compared to the start of the experiment in animals treated with Mg BT™ (*p* = 0.0255) and Mg Oxi (*p* = 0.033). At the same time, the serum Mg concentration in Mg BT™-treated animals was higher than in control animals (*p* = 0.035). In animals treated with Mg Cit and Mg Gly, there were no significant changes in the serum Mg concentration.

### 2.3. Magnesium Concentration (mmol/L) in Urine

Magnesium concentrations (mmol/L) in urine are presented in Table 1. Urinary Mg concentrations showed a treatment-related pattern similar to that observed for serum Mg concentrations. However, at the end of treatment, the Mg concentration was significantly lower in the Mg Oxi (*p* = 0.0466), Mg Cit (*p* = 0.018) and Mg Gly (*p* = 0.0079) groups compared to the start of the experiment, as well as in the Mg Cit (*p* = 0.0328) and Mg Gly (*p* = 0.0098) groups compared to the control group. There were no significant changes in the urinary Mg concentration in the Mg BT™ group, either compared with the beginning of the experiment or with the control group. In contrast to serum, where the Mg concentration increased, after 30 days of treatment, a decrease in urine Mg concentrations was observed in the corresponding groups compared to the control group.

### 2.4. Magnesium Concentration (mmol/L) in Feces

Magnesium concentrations (mmol/L) in feces are presented in Table 1. There were no changes in the Mg concentration in feces at the beginning of the experiment in any of the groups. Increased excretion of Mg at the end of the experiment was detected in the Mg BT™ group (*p* = 0.0355).

### 2.5. Magnesium Concentration in the Cerebrospinal Fluid (CSF) and in Different Brain Regions

Magnesium concentrations (mmol/L) in the CSF are presented in Table 2. The Mg concentrations in the cerebrospinal fluid (CSF) are relatively uniform, ranging from 0.67 ± 0.01 (Mg Cit and Mg Gly) to 0.71 ± 0.01 (Control). There are no statistically significant differences between the investigated groups of animals. However, a slight, non-significant increase in Mg^2+^ was observed following supplementation with Mg Gly, ranging from 11 to 17% across all brain structures. In contrast, Mg BT™ administration did not alter Mg concentrations in any of the analyzed brain regions compared to the control group.

### 2.6. Selected Biochemical Analyses in the Serum of Animals

Selected serum biochemical parameters after 30 days of treatment are summarized in the Supplementary Material (Table S4). Relative to the control group, the Mg BT™ group exhibited significantly elevated levels of albumin, cholesterol, glucose, and total protein. A significant increase in glucose was also observed in the Mg Cyt group. Alanine aminotransferase (ALT) activity was elevated in the Mg Gly group, whereas aspartate aminotransferase (AST) activity was significantly higher in both the Mg Oxi and Mg Gly groups (*p* < 0.05).

Overall, the majority of measured parameters remained within the established reference ranges. However, the albumin, AST, and total protein values exceeded the upper limits of the reference intervals. The reference ranges applied in this study were derived from the database of our Unit for Experimental Animals.

### 2.7. Oral Glucose Tolerance Test—OGTT

The results of the oral glucose tolerance test in Wistar rats after three weeks of treatment with Mg supplements are shown in Table 3. As indicated in Table 3, the initial blood glucose levels of all groups before glucose administration were equal. After oral glucose loading (2 g/kg) in both the control and test groups, blood glucose levels peaked at 30 or 60 min and then decreased over time. The blood glucose concentration measured at 120 min returned to baseline in the control and Mg Oxi groups, while in the Mg Cit, Mg Gly and Mg BT™ groups, baseline levels were reached at 180 min post-glucose overload.

Morphological characteristics of the glucose curve during an OGTT (time to peak and shape) may reflect different phenotypes of insulin secretion and action. Glucose response curves during an OGTT are shown in the Supplementary Material (Figure S1). Mg supplementation did not change the monophasic shape of glucose response curves.

**Figure 1:**
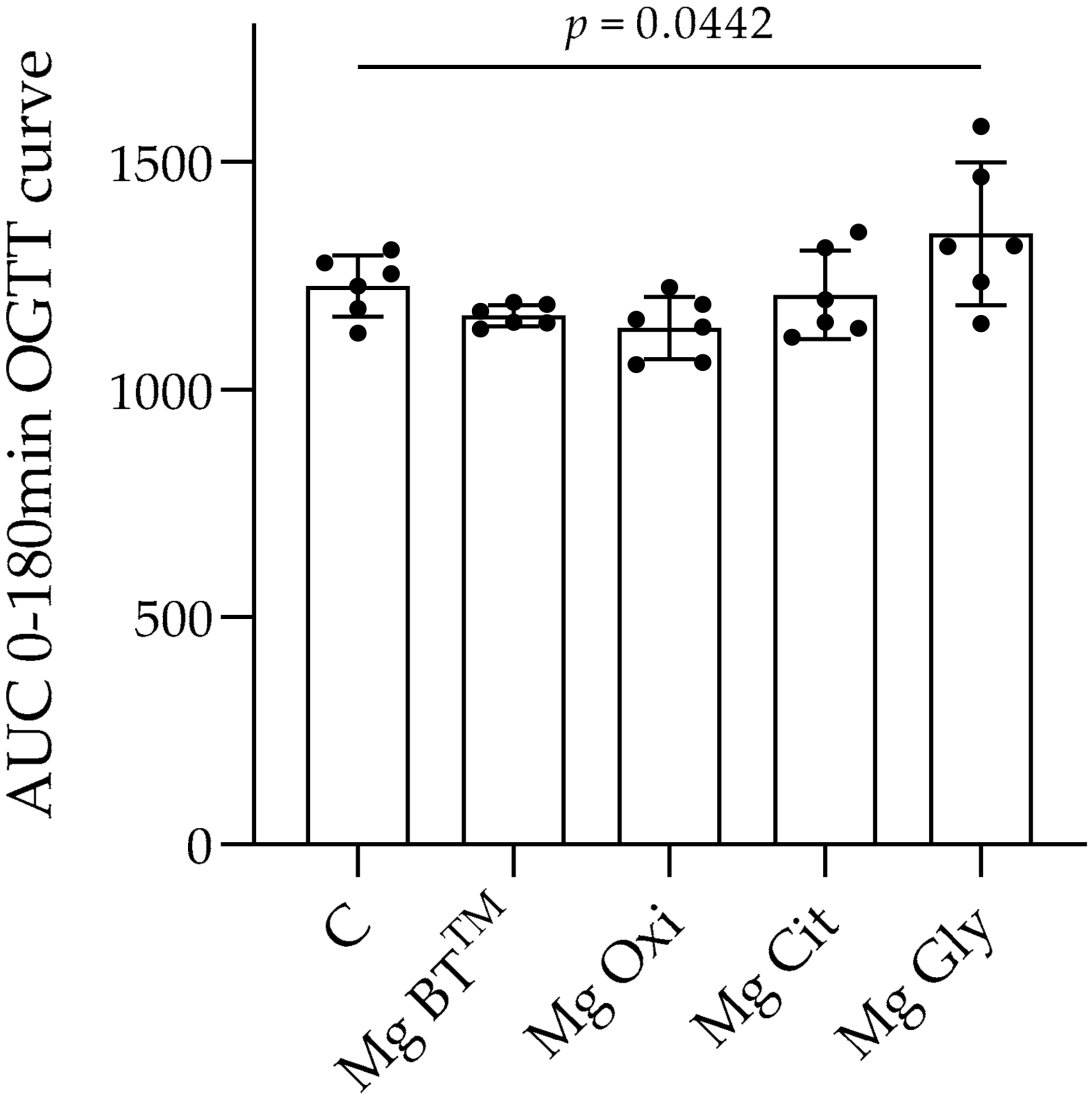
Area Under the Curve (AUC) of OGTT in the control and Mg-treated groups. Data are expressed as Mean ± standard deviations (SDs). Statistically significant at *p* < 0.05. ANOVA effects: F = 3.3885, df (5,30), *p* = 0.0351.

The results for AUC, presented as a column bar graph in Figure 1, show a statistically significant increase in the Mg Gly group compared to the control group (*p* = 0.0442). The lowest level of AUC was detected in the Mg Oxi group, but this difference was not statistically significant compared to the control group. The AUC results show the highest value in the Mg Gly group and the lowest in the Mg Oxi group.

### 2.8. Expression of Synaptic Proteins in the Cortex

For molecular analyses, tissue samples from the prefrontal cortex were collected. To investigate the potential influence of different Mg supplements on the expression of proteins included in synaptic plasticity, we performed Western blot analysis of the levels synaptophysin (SYPH), as presynaptic, and PSD-95 and drebrin, as postsynaptic markers (Table 4).

A one-way ANOVA revealed a significant effect of treatment on cortical SYPH protein levels (*p* < 0.0001) (Table 4). Post hoc analysis showed that supplementation with Mg BT™ decreased the SYPH relative abundance by 24% (*p* = 0.002) compared to the control group. Supplementation with Mg Oxi, Mg Cit, and Mg Gly did not significantly affect SYPH levels relative to controls. Moreover, SYPH levels in the Mg BT™ group were significantly lower than those in the Mg Oxi (*p* = 0.0018), Mg Cit (*p* < 0.0001) and Mg Gly (*p* = 0.0005) groups.

We evaluated the expression of total PSD-95 and its phosphorylated form (p-PSD-95), the latter serving as a marker of PSD-95 activation. There was no significant effect of treatment on total PSD-95 protein levels (*p* = 0.0678) (Table 4). Assessment of PSD-95 activation, expressed as the p-PSD-95/PSD-95 ratio, also indicated a significant treatment effect (*p* = 0.0124). Post hoc analysis identified a significant increase in the Mg Oxi group compared with the control for both Phospho-PSD-95 (*p* = 0.0223) and PSD-95/phospho-PSD-95 (*p* = 0.0452), respectively (Table 4). Although other supplemented groups showed a 30–40% elevation in PSD-95 activation, these changes did not reach statistical significance.

A one-way ANOVA revealed a significant effect of treatment on drebrin protein levels (*p* = 0.0049). Post hoc analysis showed that supplementation with Mg Cit and Mg Gly significantly increased drebrin expression by 96% (*p* = 0.0218) and 89% (*p* = 0.0383), respectively, compared to the control group. Mg Oxi supplementation induced a non-significant increase of 38%, while drebrin levels in the Mg BT™ group did not differ from the control.

### 2.9. Expression of the NR2B and BDNF Isoform X in the Cortex

PCR analysis was performed to evaluate the expression of genes encoding the NR2B subunit of the N-methyl-D-aspartate (NMDA) receptor and the brain-derived neurotrophic factor (BDNF), both of which have previously been reported to be influenced by magnesium administration.

**Figure 2:**
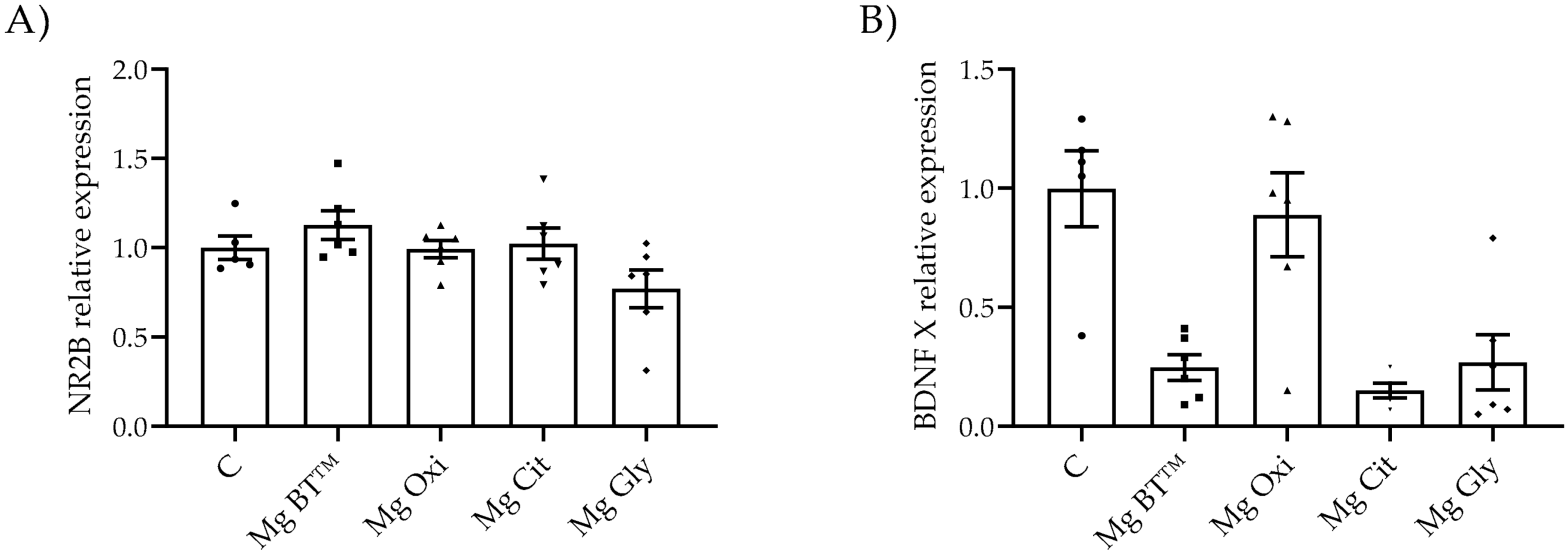
Expression of NR2B and BDNF isoform X expression in the cortex of control and the animals administered magnesium supplementation. The data are expressed as relative values of mRNA expression (mean ± SEM) relative to the respective controls. Kruskal–Wallis statistics: (**A**) 7.361 (5,29), *p* = 0.1180; (**B**) 15.591 (5,28), *p* = 0.056.

The Kruskal–Wallis test revealed no significant effect of treatment on NR2B gene expression. None of the magnesium supplements significantly altered NR2B mRNA levels in the cerebral cortex compared to the control group or among each other (Figure 2A).

Based on previous findings, we analyzed the expression of the BDNF isoform transcribed from exon X (Figure 2B). Primers for BDNF isoform X were designed to span a region common to all isoforms (I–X), which is critical for mRNA localization, translation, and synaptic stability of the BDNF protein.

The Kruskal–Wallis test did not indicate a significant effect of treatment on BDNF isoform X mRNA expression. Although a general decrease in BDNF X expression was observed in the Mg BT™, Mg Cit and Mg Gly groups, Dunn’s multiple comparison post hoc test did not show significant differences (Figure 2B).

### 2.10. Locomotor Behavior in the Open Field Test

**Figure 3:**
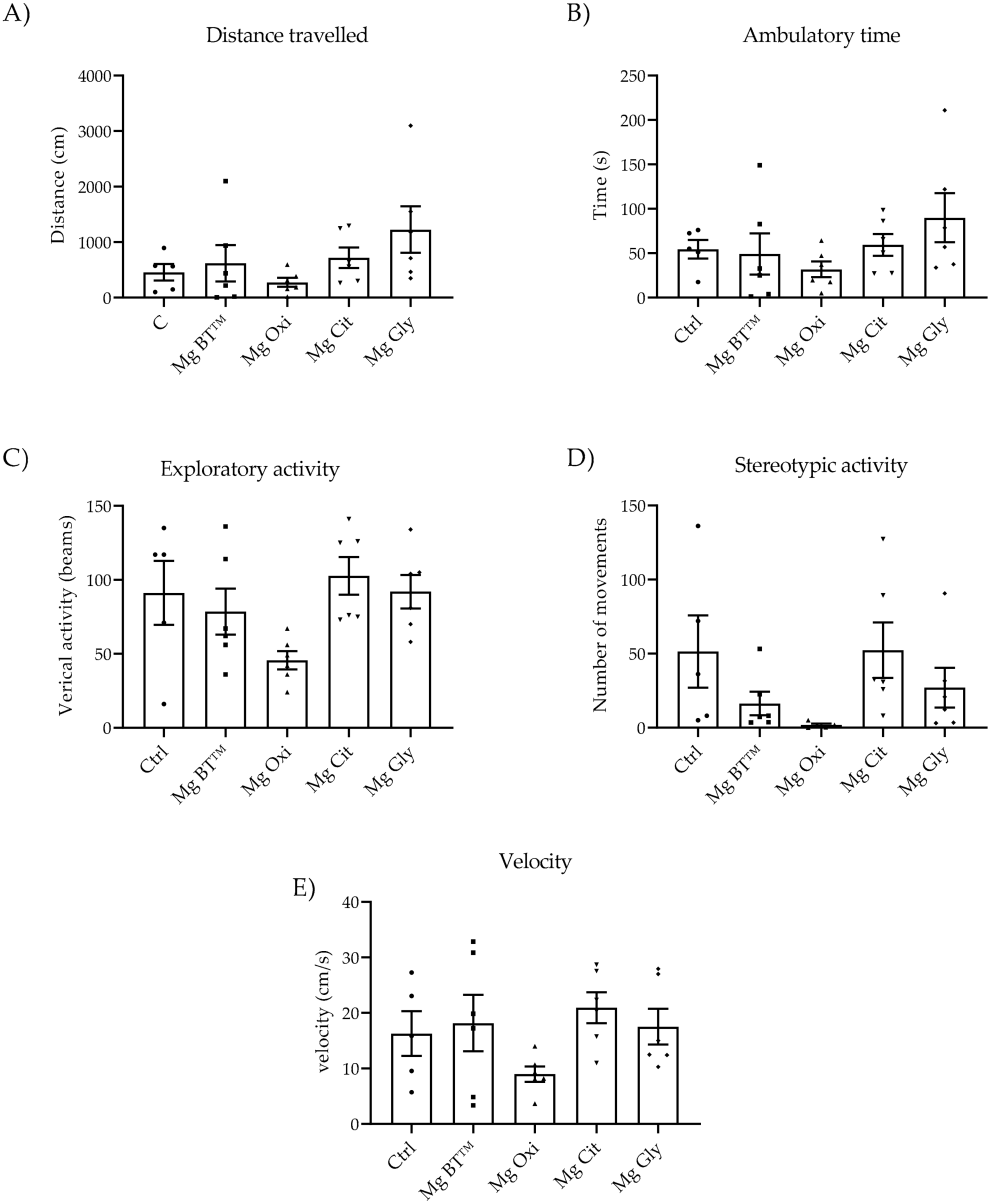
General locomotor activity in the open field arena. Open Field test parameters (total distance traveled (DT); total duration of movement–ambulatory time (AT); exploratory activity, i.e., vertical activity (V1B); stereotypic behavior and velocity of movement (DT/AT)) of animals treated with different Mg supplements. ANOVA effects: (**A**) F = 1.806, df = 28 (4,24), *p* = 0.1607; (**B**) F = 1.345, df = 28 (4,24), *p* = 0.2823; (**C**) F = 2.626, df = 28 (4,24), *p* = 0.0597; (**D**) F = 2.014, df = 27 (4,23), *p* = 0.1260; (**E**) F = 1.696, df = 28 (4,24), *p* = 0.1839.

One-way ANOVA showed no statistically significant effect of magnesium supplementation on these parameters in any treatment group. However, several notable trends were observed. Animals supplemented with Mg Oxi consistently exhibited a markedly lower level (~40–50%) of motor activity compared to controls, while Mg-glycinate-treated animals showed a mild increase in activity. Animals receiving Mg BT™ displayed locomotor parameters within the control range (Figure 3A,B,E).

A similar decline (>50%) in the Mg Oxi group was also observed in rearing behavior (vertical activity), an indicator of exploratory behavior, as well as in grooming behavior, which reflects repetitive and stereotypic activity. Interestingly, a reduction in stereotypic activity was recorded across all magnesium-treated groups, including Mg BT™ (Figure 3D).

### 2.11. Anxiety-Related Behavior

Anxiety-like behavior was assessed by measuring the time spent in the central zone of the open field arena and through complementary testing in the light–dark (LD) box.

**Figure 4:**
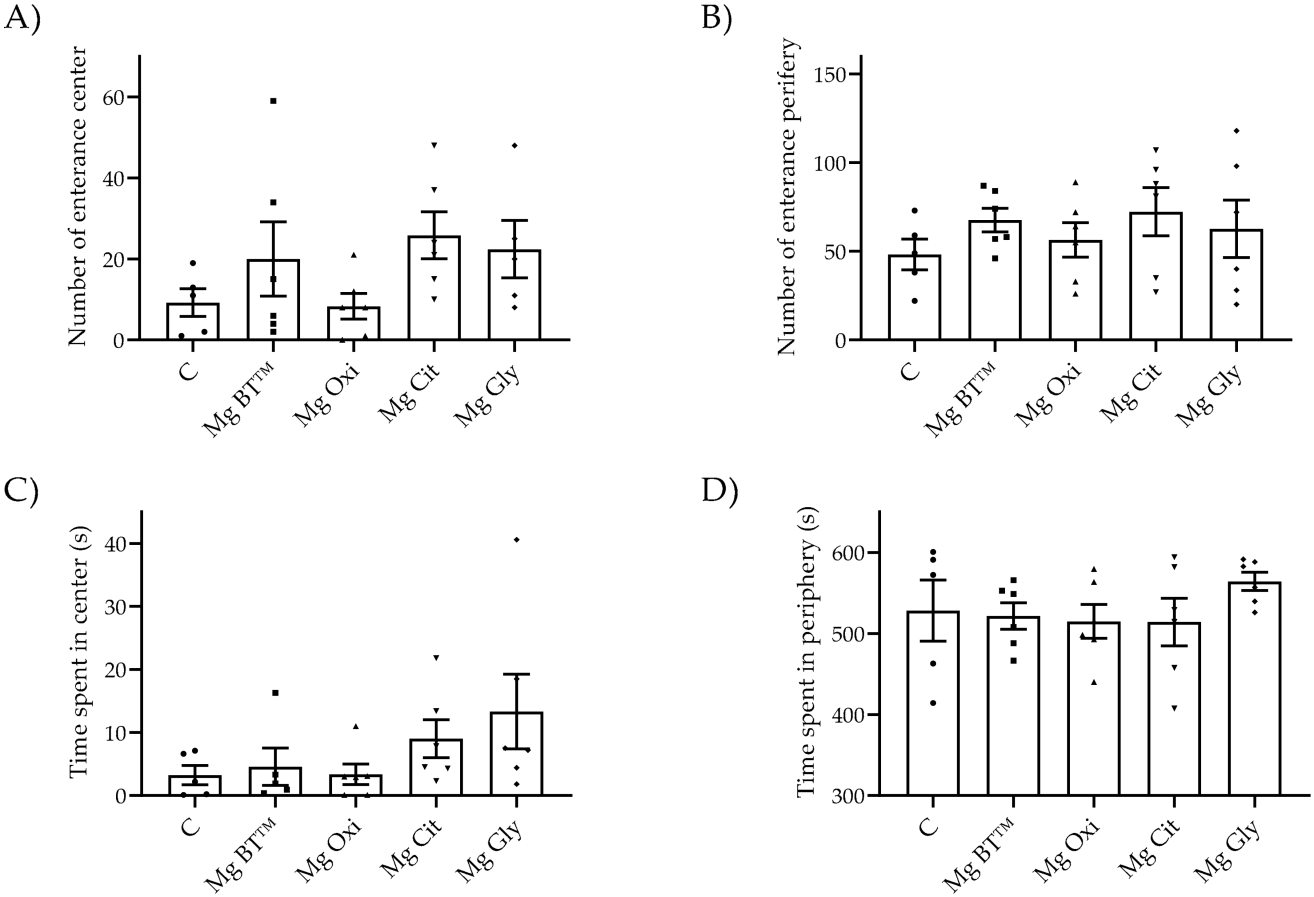
Open Field anxiety-like behavior (number of entries and time spent in the central quadrants versus the peripheral quadrants) of animals treated with different Mg supplements. Kruskal–Wallis statistics: (**A**) 7.409 (5,28), *p* = 0.1158; (**B**) F = 0.6161, df = 28 (4,24), *p* = 0.6553; (**C**) 7.589 (5,28), *p* = 0.1079; (**D**) 3.832 (5,29), *p* = 0.429.

Increased time and frequency of entries into the central area of the open field indicate reduced anxiety. Animals supplemented with Mg Cit and Mg Gly showed more than a twofold increase in the number of entries and a 2–4-fold increase in time spent in the central zone compared to controls, although these differences did not reach statistical significance by one-way ANOVA. After Mg BT™ supplementation, only the number of center entries was slightly increased compared to the control (Figure 4).

**Figure 5:**
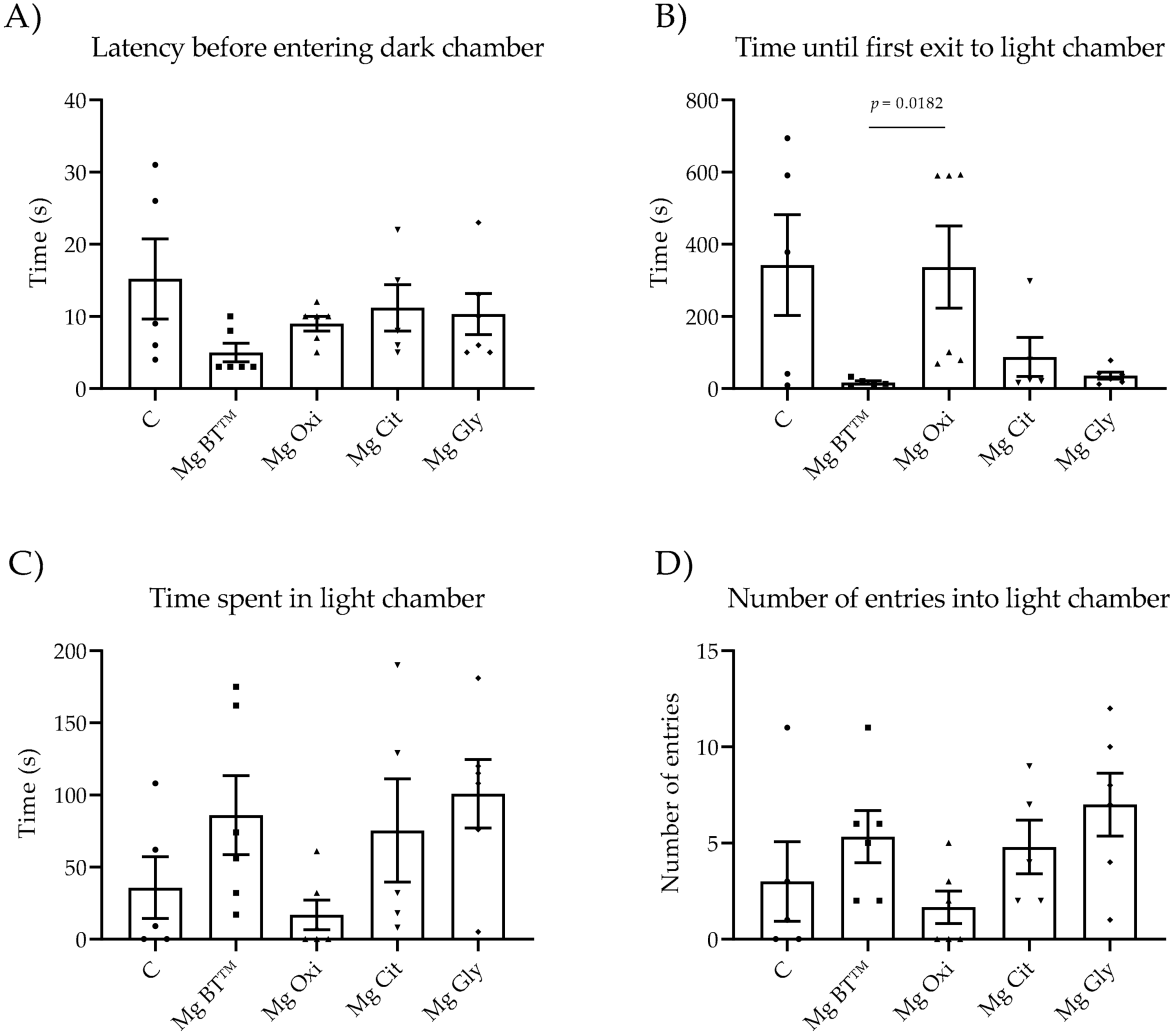
Light–dark box test (latency before entering the dark chamber, time until first exit into light chamber, time spent in the light chamber and number of entries into light chamber) of animals treated with different Mg supplements. Kruskal–Wallis statistics: (**A**) 5.026 (6,34), *p* = 0.4128; (**B**) 14.95 (6,32), *p* = 0.0106; (**C**) F = 1.375, df = 33 (5,28), *p* = 0.2635; (**D**) F = 1.421, df = 33 (5,28), *p* = 0.2472.

Similarly, no statistically significant effects of magnesium supplementation were observed in the LD chamber test. Nonetheless, animals treated with Mg BT™ showed shorter latencies to enter the dark compartment, significantly shorter intervals before re-entering the light compartment (*p* < 0.05), more frequent transitions and longer time spent in the illuminated zone, which suggests reduced anxiety and greater comfort in the light environment (Figure 5). In contrast, Mg Oxi-treated animals exhibited the opposite trend, indicating increased anxiety-like behavior.

### 2.12. Histological Analysis of the Liver and Kidneys

Morphological assessment of the liver and kidney (size, color, surface appearance, presence of lesions, and consistency) showed no evidence of pathology or structural alteration after treatment with Mg BT™ and other Mg forms compared to the control. Therefore, in this paper, we present only micrographs of control and animals treated with Mg BT™.

#### 2.12.1. Histological Analysis of the Liver

**Figure 6:**
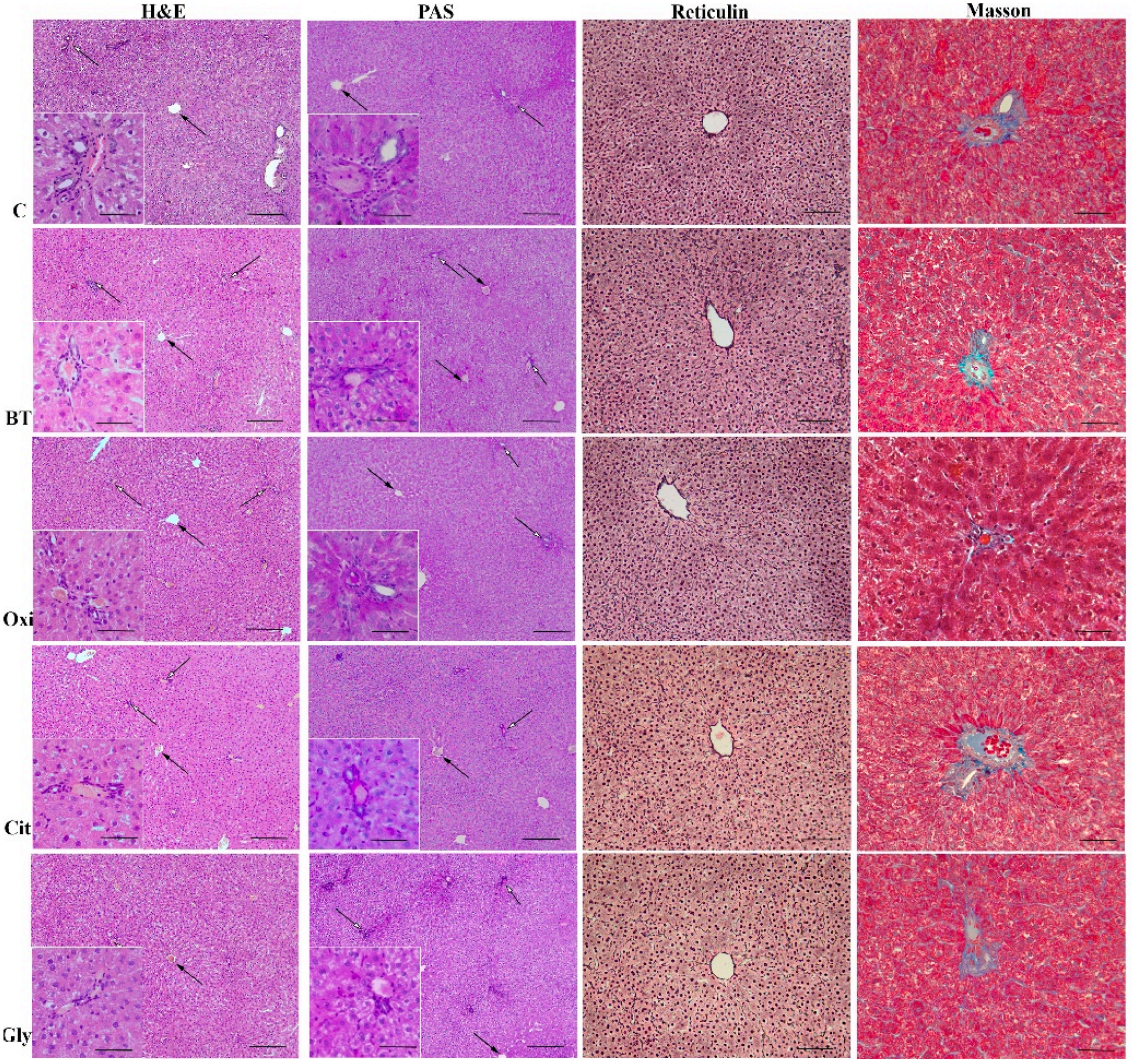
Representative micrographs of liver sections from control animals (C) and animals treated with Mg BT^™^ (BT), Mg Oxi (Oxi), Mg Cit (Cit) and Mg Gly (Gly). H&E and PAS staining demonstrate the overall hepatic architecture at low magnification (10× objective; scale bar = 200 µm), with higher-magnification insets (40× objective; scale bar = 50 µm) showing the portal triads in greater detail. Representative reticulin- and Masson’s trichrome-stained sections are presented at 40× objective magnification (scale bar = 50 µm), highlighting the central vein and portal triads, respectively. Black arrows indicate central veins, whereas white arrows indicate portal triads.

In control animals, H&E staining of the liver showed normal lobular organization, with a central vein surrounded by portal triads at the corners of each lobule. Each triad consisted of a hepatic artery, portal vein, and bile duct. Hepatocytes are polygonal cells with centrally located nuclei, occasionally binucleated, and are arranged in radial cords extending from the central vein. Sinusoidal capillaries separated the hepatocyte cords, allowing efficient exchange between blood and parenchyma. Cytoplasmic eosinophilia varied according to mitochondrial and smooth endoplasmic reticulum content, but most hepatocytes exhibited strong eosinophilia. After treatment with Mg BT™, the hepatic architecture remained normal and comparable to controls, with preserved lobular structure and typical hepatocyte morphology. Similarly, no histopathological alterations were observed following administration of the three individual Mg forms, Mg Oxi, Mg Cit, and Mg Gly. In all treated groups, the liver structure corresponded to that of the controls, exhibiting normal lobular architecture and characteristic microscopic features of hepatocytes (Figure 6).

In the liver of the control group, PAS staining revealed that glycogen distribution is generally uniform across the liver parenchyma. PAS-positive staining intensity is slightly more pronounced in the periportal zones around the portal triads and somewhat less intense in the centrilobular hepatocytes near the central vein. In the experimental Mg BT™ group, the distribution of glycogen matches the histological profile of the control livers, and a similar distribution pattern is observed after treatment with other Mg forms, indicating preserved hepatic glycogen distribution (Figure 6).

After applying Reticulin silver staining techniques, reticulin fibers in the control group appeared as black-to-dark brown fibers, revealing a fine network that supports the architecture of the liver. The reticulin network remained unaltered after treatment with Mg BT™ and other Mg forms (Figure 6).

Mason’s trichrome staining in the control group showed blue collagen fibers, which form a highly organized and delicate network primarily composed of type I and III collagen. These fibers are mainly localized around the central vein and portal triads, while the perisinusoidal space (Space of Disse) contains minimal collagen, providing structural support to hepatocytes and sinusoidal endothelial cells. Normal organization of connective tissue was observed after administration of Mg BT™ and four other forms of Mg, indicating no signs of liver pathology such as fibrosis, hepatitis, or cirrhosis (Figure 6).

#### 2.12.2. Histological Analysis of the Kidneys

**Figure 7:**
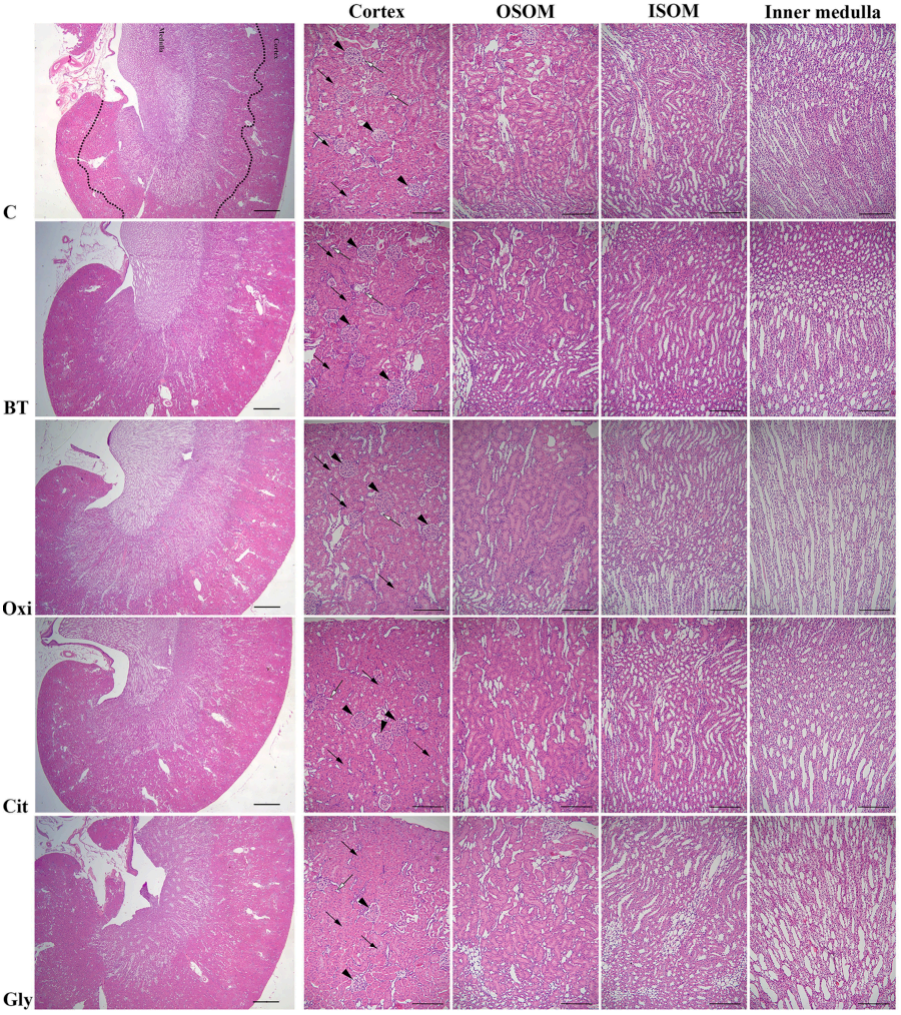
Representative micrographs of kidneys of control animals (C) and animals treated with Mg BT^™^ (BT), Mg Oxi (Oxi), Mg Cit (Cit) and Mg Gly (Gly). HE staining revealed the basic histological zonation of the kidney, distinguishing the cortex and medulla—left column; bar = 1000 µm. OSOM—outer stripe of outer medulla zone; ISOM—inner stripe of outer medulla zone; arrowheads—renal corpuscles; black arrows—proximal tubules; white arrows—distal tubules; bar = 200 µm.

The zonal distribution of the kidney, comprising the outer cortex and the inner medulla, which is further subdivided into outer and inner stripes that merge into a single renal papilla, was clearly distinguishable in cross-sections following H&E staining. The overall histological structure of the kidneys remained unaltered after the administration of Mg BT™ and other Mg forms (Figure 7).

**Figure 8:**
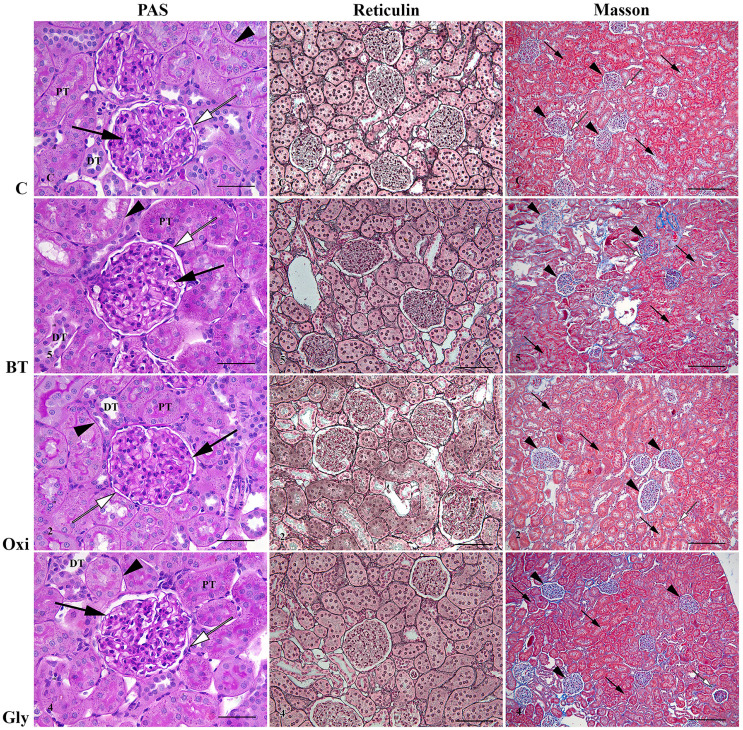
Representative micrographs of kidneys of control animals (C) and animals treated with Mg BT^™^ (BT), Mg Oxi (Oxi), Mg Cit (Cit) and Mg Gly (Gly). PAS staining showed basement membrane of proximal tubules—arrowheads; glomerular basement membranes—black arrows; Bowman’s capsule—white arrows; proximal tubule—PT; distal tubule—DT; bar = 50. Masson’s trichrome-stained sections of kidneys: renal corpuscles—arrowheads; proximal tubules—black arrows; distal tubules—white arrows; bar = 200 µm. Reticulin-stained sections of kidneys revealed reticular fibers forming a delicate, black network around the renal corpuscles and tubules; bar = 100 µm.

In the kidneys, PAS staining is particularly useful for visualizing the basement membrane and glomerular architecture and is therefore used to detect membranous nephropathy and other glomerular diseases. Normal morphology with intact Bowman’s capsules, without mesangial expansion or hypercellularity, was observed, indicating a healthy glomerular structure. The renal tubules also appeared normal, with a well-defined brush border in the proximal tubules and no dilatation or atrophy. Renal vasculature was intact, with no evidence of hyalinosis or sclerosis. Detailed histological analysis showed that in the Mg BT™ group, renal corpuscles appeared intact and well defined, and glomeruli showed no signs of sclerosis or inflammation (Figure 8).

Reticulin staining is a specialized histopathological technique primarily used to visualize reticulin fibers, which are composed of type III collagen. This stain is particularly valuable in assessing the structural integrity of basement membranes, including those of glomeruli and tubules. The analysis revealed that the architecture of the kidneys in the control, Mg BT™ and other Mg-treated groups was well preserved, with no pathological changes detected. The reticulin fibers exhibited a normal pattern, indicating intact structural support within the kidney parenchyma. The fibers were uniformly distributed around the glomeruli and tubules, suggesting no loss of structural integrity or fibrosis (Figure 8).

Mason’s trichrome staining differentiates collagen (blue) from other tissue components and is particularly useful for assessing fibrosis, sclerotic changes, or mesangial matrix expansion. Histological analysis of the control group revealed collagen fibers predominantly distributed around Bowman’s capsule, renal tubules, blood vessels, and in the interstitial spaces. After treatment with Mg BT™ and other Mg forms, the glomerular, tubular, interstitial, and vascular components showed normal renal morphology without any signs of fibrosis or inflammation, suggesting normal kidney function (Figure 8).

**Figure 9:**
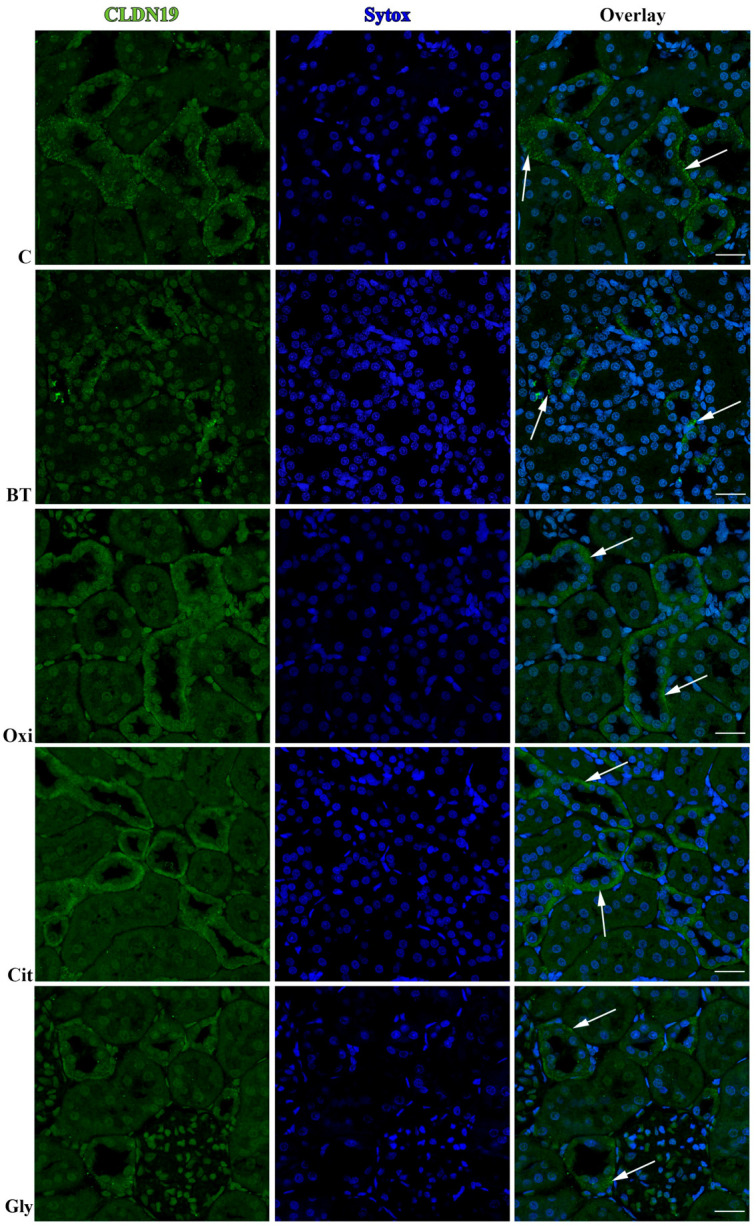
Representative confocal micrographs of Claudin-19 (green) in kidney of control animals (C) and animals treated with Mg BT^™^ (BT), Mg Oxi (Oxi), Mg Cit (Cit) and Mg Gly (Gly); white arrows—Claudin-19-positive tubules: bar = 25 µm.

Immunofluorescence analysis of Claudine-19 in the kidney (CLDN-19) shows that it is a multifaceted tight junction protein essential for renal electrolyte homeostasis. In control kidneys, a characteristic mosaic pattern of CLDN-19 expression in the thick ascending limb of Henle’s (TAL) can be seen in the cortical and medullary parts. The intensity and pattern of CLDN-19 expression in the Mg BT™ group were similar to those in the controls, although occasional intense fluorescent signals were noted (Figure 9).

## 3. Discussion

The aim of the present study was to compare the effects of Mg BT™, which is a blend of several forms of Mg, with widely used Mg supplements on selected biological parameters in rats. Magnesium deficiency may result from decreased intake, absorption, internal redistribution or increased loss of Mg. Magnesium deficiency can cause serious biochemical changes, as it plays an essential role in a wide range of fundamental biological processes [17]. Magnesium deficiency is also linked to a wide range of chronic health conditions, such as hypertension, cardiovascular disease, diabetes, osteoporosis and mood disorders [17]. To avoid the risk of Mg deficiency, supplements of this mineral are often recommended. Conventional Mg supplements contain one of two distinct sources of elemental Mg: inorganic or organic salts of Mg [18]. Inorganic salts provide a high amount of elemental Mg but have very limited bioavailability due to their poor solubility. Organic sources of Mg offer high solubility but provide lower levels of elemental Mg [19]. Studies on the bioavailability of different Mg salts consistently show that organic salts of Mg have higher bioavailability than inorganic salts [20]. The literature on the bioavailability of various Mg forms provides limited information on the optimal Mg salt for animal and human supplementation. Magnesium is essential for many physiological functions in mammals. The dietary requirement for adequate rat nutrition depends on several factors that affect Mg availability, the most important being the amounts of dietary calcium, phosphorus, and vitamin D [21]. In humans, only a few studies have examined the bioavailability of different Mg salts, and these studies are based on a limited number of Mg preparations, making it difficult to determine clearly which Mg salt has the best bioavailability [15].

Body weight was monitored throughout the experiment, since changes in it often reflects alterations in metabolism, appetite, or systemic toxicity, and it represents one of the most sensitive indicators of the general health and safety of administered compounds to animals. In the present study, some variability in initial body weight was observed among experimental groups. However, this is a common feature in rodent studies and is unlikely to have influenced the outcomes, as all animals were dosed according to body weight (mg/kg) with daily adjustment of supplementation. Therefore, systemic exposure to Mg was normalized across groups. Furthermore, no evidence was observed in our study that initial body weight was associated with differences in body weight gain, suggesting that the reported effects are primarily attributable to the administered magnesium formulations rather than baseline variability. Relative liver weight, as a size-corrected functional index of the organ, was lower in animals treated with Mg Oxi, while relative left kidney weight was lower in almost all experimental groups with respect to control animals. The relationship between Mg intake and body mass gain or body mass index (BMI) has been investigated, but research to date indicates that this association remains unclear. In a large cohort (CARDIA study, USA), an inverse correlation was found between Mg intake and the risk of developing obesity over approximately 30 years: people with higher Mg intake had a lower risk of obesity [22]. Some other investigations reported an inverse association between Mg intake and BMI associated with lower serum glucose levels and with lower waist circumference (WC) [23]. However, research by Rafiee et al. (2021) [24] showed that supplementation did not significantly change body weight, BMI or the percentage of body fat in individuals of normal weight, but it was the opposite for obese individuals, where supplementation significantly reduced WC. Magnesium may indirectly reduce fat accumulation and body mass gain by improving insulin function, reducing inflammation and enhancing glucose metabolism. Additionally, dietary Mg intake is often linked to a healthier diet (including more vegetables, whole grains and nuts), making it difficult to distinguish the effect of Mg itself from that of the overall diet [23]. In supplementation studies, there are large differences due to the use of different doses of Mg, varying initial statuses (low or normal Mg levels) and different populations (age, sex, health), resulting in varied outcomes. Under the conditions of the present study, Mg BT™ demonstrated a favorable safety profile comparable to that of the other tested magnesium preparations.

Several animal studies have compared the bioavailability of different Mg salts and generally reported superior absorption of organic forms compared with inorganic preparations. Lindberg et al. (1990) [14] demonstrated that magnesium citrate exhibited higher bioavailability than magnesium oxide, while Schuette et al. (1994) [16] reported favorable absorption characteristics of magnesium glycinate compared with inorganic Mg salts. Similarly, Firoz and Graber (2002) [25] showed that the bioavailability of Mg preparations is strongly influenced by their solubility and chemical form. Although our study was not designed to directly quantify Mg absorption, the increased fecal Mg excretion observed in supplemented animals, together with the absence of adverse effects on liver and kidney histology, indicates effective handling of the administered Mg loads. Furthermore, the comparable biological responses observed among the tested Mg preparations suggest that all investigated forms were capable of maintaining systemic Mg homeostasis under the conditions of the present study.

After absorption, Mg is transported via the bloodstream to various tissues and organs. Transfer of Mg from serum to urine begins immediately when Mg pools are saturated. Magnesium in the body is primarily found in bone (53%) and soft tissues (46%), with the remaining 1% in the blood, either in the free ionized form (54–65%) or bound to proteins (27–34%) or to anions (8–12%). Only the free, ionized form of Mg is physiologically active. In serum, reference values for ionized Mg in rats range from 0.75 to 0.95 mmol/L, and similar values are found in the CSF [26]. Serum Mg concentration is not an adequate indicator of Mg requirements because only 1% of body Mg is found in serum. Some studies have reported that serum Mg enters the CSF, but the ionic composition of the CSF remains constant during changes in the blood ionic composition in humans and animals under both normal and pathological conditions [27]. To maintain optimal intracellular and extracellular magnesium levels, Mg homeostasis is regulated by hormonal and non-hormonal mechanisms. Parathyroid hormone and calcitriol (active vitamin D) play key roles in Mg homeostasis. Parathyroid hormone increases renal Mg reabsorption in response to low serum Mg levels, while calcitriol enhances intestinal Mg absorption [6]. When Mg stores in the body are saturated, the body attempts to maintain normal Mg homeostasis by increasing excretion, decreasing reabsorption in the renal tubules and increasing excretion through the feces. In accordance, increased excretion of Mg via feces in Mg-treated rats in our study suggests elimination of unabsorbed Mg. The brain has two main barrier systems: the blood–brain barrier (BBB), formed by brain capillary endothelial cells, which separates the blood from the extracellular fluid, and the blood–CSF barrier, formed by choroidal epithelial cells, which separates the blood from the CSF. Transport through the BBB is highly regulated, and an increase in blood Mg does not necessarily result in a significant increase in the brain [28,29]. Some forms of Mg (such as citrate, oxide, glycinate, malate, etc.) have better absorption in the body in terms of serum and intestinal levels, but there is insufficient evidence that they significantly increase Mg levels in the brain or CSF. It is known that organic forms are better than inorganic forms of Mg, but this does not focus entirely on BBB crossing [1]. It is assumed that under supplementation with physiological doses of Mg, as in our experiment, its concentration in the CSF does not change significantly. Reference values for rat serum biochemical analyses are rarely universal. It is important to rely on the specific control values for the group of rats used in a particular study, rather than on published general reference ranges. It is also known that the values of some analyses can vary significantly depending on many factors [30]. With this in mind, a certain trend was observed among the recorded changes in biochemical parameters: albumin, cholesterol, and total proteins were increased compared to the control values. All Mg-treated animals had increased serum AST activity above reference values, and rats treated with Mg Oxi and Mg Gly had increased AST activity compared to control animals. However, based on the unchanged histological structure of the liver and associated biochemical parameters, such as ALT, ALP and CK, it seems that Mg supplementation did not induce liver damage.

Notably, the most attention is drawn to the increase in the glucose concentration observed in rats treated with Mg BT™ and Mg Cit compared to the control rats. However, this increase remains within the reference range and cannot be considered hyperglycemia. Magnesium supplementation has been linked to various aspects of blood glucose regulation [31]. Some studies suggest that Mg plays an important role in maintaining healthy blood sugar levels and insulin sensitivity. It has been shown that Mg acts as a second messenger for insulin action, and insulin is a regulatory factor for intracellular Mg accumulation. Conditions associated with insulin resistance, such as hypertension or aging, are also linked to low intracellular Mg levels [32]. Magnesium supplementation may help reduce the risk of developing type 2 diabetes by improving how the body processes glucose. Some studies indicate that Mg intake can reduce fasting blood glucose levels and HbA1c (a marker of long-term blood sugar control) [33]. Magnesium is known to normalize the glucose concentration in rats with streptozotocin-induced type 1 diabetes, but it appears to have a different mechanism of action on the glucose concentration in healthy animals [34]. At the same time, it is also known that even low concentrations of anesthetic gases can alter the glucose concentration in rats. There is evidence suggesting that isoflurane can affect the serum glucose level in rats, though the precise mechanisms may vary based on factors such as dosage, exposure time and the specific rat strain. In this way, reference values can be shifted to a higher level between 8.2 and 11.6 mmol/L [35]. In general, isoflurane can activate the stress response in animals, leading to an increase in circulating catecholamines (such as epinephrine), which can stimulate glycogenolysis (breakdown of glycogen into glucose) and gluconeogenesis (production of glucose from non-carbohydrate precursors), potentially raising blood glucose levels. Isoflurane may induce temporary insulin resistance, which could further contribute to elevated blood glucose levels during and after anesthesia. However, it is generally expected that isoflurane exposure could lead to transient increases in serum glucose due to the physiological stress and metabolic changes it induces [35]. To better understand the impact of Mg supplements on glucose metabolism, we conducted an OGTT. The OGTT results show the time to glucose peak during the test and identify the prediabetes risk. The shape of the glucose curve can be characterized as biphasic or monophasic [36]. Curves are classified as monophasic if glucose reaches a maximum between 30 and 90 min, followed by a decrease until 120 min. Curves are classified as biphasic if glucose peaks at 30 or 60 min, followed by a nadir and a second peak by 120 min. Mg supplementation did not change the monophasic shape of glucose response curves, except in the Mg Gly group, where it was increased with respect to control animals. Based on these findings, we can state that Mg from the tested Mg supplements have a positive effect (Mg Oxi), no effect (Mg Cit, Mg BT™), or a negative effect (Mg Gly) on glucose metabolism. In conclusion, Mg supplementation may help improve blood glucose control, but due to the use of anesthetics, the data obtained were shifted towards higher values.

Magnesium plays an important role in regulating synaptic plasticity, leading to improved learning and memory, with an increased number of presynaptic release sites, greater density of synaptic markers and stronger synaptic plasticity via NR2B-containing NMDA receptors [37]. Synaptophysin is a well-established presynaptic marker and a structure-related protein localized within neurotransmitter-containing vesicles expressed in both excitatory and inhibitory neurons [37]. To our surprise, we detected a slight (~20%) non-significant decrease in the Mg BT™ group. We assume that this reduction in synaptophysin is not indicative of synapse loss but might reflect a homeostatic adjustment. Namely, it has been shown that Mg supplementation can promote inverse synaptic regulation, where strengthening of some synapses may lead to the weakening or elimination of neighboring synapses [37], which is an essential principle for maintaining neuronal circuit stability and brain function. Post-synaptic density protein-95 (PSD-95) is a key scaffolding protein and a marker of excitatory synapses. It directly binds to the NR2 subunits of glutamatergic receptors (e.g., NMDAR, AMPAR) and other synaptic receptors, thereby stabilizing excitatory synaptic transmission and enhancing synaptic strength [38]. Expression of PSD-95 and drebrin, which is a marker of synaptic spines, did not decline, suggesting that postsynaptic structural integrity is maintained despite a modest decrease in presynaptic activity. A study by Zhou et al. (2024) [39] showed that Mg from Mg Thr in rats alters the synaptic configuration in the hippocampus, and a difference is observed in the level of PSD-95 at individual synapses. Increasing brain Mg levels in aging animals restores synaptic configurations similar to those of young animals, coinciding with improved learning and memory. These findings establish intracellular Mg as a crucial factor in reconfiguring synaptic connectivity at dendrites, thereby optimizing their branch-specific properties in information processing [39]. However, evidence for direct regulation of phosphorylated PSD-95 is very limited or absent in the available literature. It is possible that Mg-mediated signaling indirectly influences PSD-95 phosphorylation if Mg alters the activity of kinases or phosphatases that act on PSD-95 (e.g., CaMKII, MAPK, GSK-3β). Drebrin is an actin-binding protein localized in dendritic spines, where it plays a crucial role in their formation, maturation, and structural plasticity, influencing both spine shape and density [40]. Decline of drebrin results in the delay of synapse formation and inhibition of postsynaptic protein accumulation, including PSD-95 and glutamate receptors, as observed in dementia and during physiological aging [40]. Preserved or increased levels of drebrin expression following Mg supplementation, as found herein, may promote synaptic plasticity, as it serves as a platform for the molecular assembly of other postsynaptic proteins, such as PSD-95 or glutamate NMDA receptors. The functional plasticity of presynaptic terminals is determined by multiple factors, one of which is the postsynaptic NR2B composition of NMDA excitatory receptors, as well as the amount of released BDNF and the sensitivity of the presynaptic terminal to the released BDNF [41]. We observed stable NR2B mRNA levels, but this does not preclude changes in NR2B protein expression or receptor activity, as even subtle shifts in the extracellular Mg concentration modulate NMDA currents under physiological conditions [6]. This could lead to suppression of BDNF transcription and altered presynaptic regulation, as BDNF modulates vesicle release and synapse maturation [41]. The NR2B (GluNR2B) subunit is a critical component of NMDA receptors that mediate glutamate-dependent synaptic plasticity, learning, and memory [42]. Magnesium is essential for the regulation of NMDA receptors, as Mg blocks the channel of this receptor when the membrane is at rest. A study by Mony et al. (2009) [43] showed that Mg is one of the allosteric modulators of NR2B receptors and therefore of synaptic plasticity, chronic pain, psychosis and related conditions. The effects of Mg are complex because it acts as an NMDA channel blocker, a regulatory ion and also affects NR2B expression and synaptic plasticity. Age-related memory loss is believed to result from reduced synaptic plasticity, including changes in the NR2B subunit composition of the NMDA receptor. Dietary supplementation with Mg is surprisingly effective in the brain in upregulating NR2B expression and improving memory in preclinical studies [44]. BDNF is an essential neurotrophic factor that regulates neuronal survival, differentiation, synaptic plasticity, neuroprotection, and cognitive function. The BDNF gene produces 10 mRNA isoforms through transcription from distinct promoters [45]. The study by Albumaria et al. (2011) [46] shows that treatment with Mg in the form of Mg Thr enhances the retention of fear memory extinction without enhancing, impairing or erasing the original fear memory. Elevation of brain Mg induced increases in NR2B-containing NMDARs, activation of NMDAR signaling, BDNF expression, and synaptic plasticity in the prefrontal cortex [46] and hippocampus [41]. In a randomized study of healthy adults aged 18–65 years, it is also shown that supplementation with Mg in the form of a Mg Thr-based formula also improved cognitive function [47].

Animals treated with Mg BT™ showed a tendency toward reduced anxiety-like behavior. Given the limited sample size and the use of a single behavioral paradigm, this observation should be considered preliminary and warrants validation in future studies involving larger cohorts and complementary anxiety assessments. However, this finding warrants repetition of the experiment with more animals per group and, if possible, the inclusion of additional anxiety tests. Most previous studies examining Mg effects have used in vitro models, while in vivo experiments have typically involved aging or disease models (such as Alzheimer’s or Parkinson’s), in which Mg deficiency is common due to dietary insufficiency or metabolic changes, resulting in pronounced benefits from supplementation [48]. In contrast, our young, healthy rats on adequate nutrition exhibited subtler effects. Although we did not include learning- and memory-specific tests, open field parameters, which reflect exploration of novel environments (distance traveled and vertical activity), revealed no significant effects of supplementation. The behavioral trends towards reduced anxiety-like behavior observed in the Mg BT™ group require replication with specific anxiety and cognitive tests to confirm potential benefits. The literature data indicate that Mg has a calming and anti-anxiolytic effect in states of induced stress. Pochwat et al. (2014) [49] showed that administration of Mg (10, 15 and 20 mg/kg) reduced hyperactivity in the open field test and that Mg had an anxiolytic effect. Magnesium-deficient mice have also been shown to display increased anxiety-related behavior in the light/dark and open field tests [50]. All Mg-treated groups show a reduction in stereotypic activity. In Mg BT™ supplementation, the number of center entries was slightly increased compared to the control. Animals treated with Mg BT™ showed shorter latencies to enter the dark compartment, shorter intervals before re-entering the light compartment, more frequent transitions and longer time spent in the illuminated zone, which suggests reduced anxiety and greater comfort in the light environment. In contrast, Mg Oxi-treated animals exhibited the opposite trend, indicating increased anxiety-like behavior.

Overall, while we observed slight decreases in synaptophysin protein following Mg BT™ supplementation, without significant changes in postsynaptic markers or NR2B mRNA, these effects may reflect a homeostatic synaptic adjustment. The concomitant decrease in BDNF mRNA further suggests coordinated regulation of presynaptic activity and neurotrophic signaling. These molecular changes occurred without significant alterations in locomotor or anxiety-related behavior but indicate subtle presynaptic modifications induced by Mg forms with higher brain bioavailability, necessitating further functional and behavioral evaluation.

Most recent studies investigating Mg and its effects on the liver and kidneys in animals show prevention or protection in models of liver and kidney disease, rather than a detrimental effect of high Mg itself [51,52]. Our study shows that supplementation with Mg in the form of Mg BT™ and other Mg supplements did not alter hepatic or renal histological structure. Most studies suggest that Mg deficiency creates preconditions for liver damage, such as steatosis, inflammation and oxidative stress, which can lead to structural changes [53]. Low Mg levels in serum and liver tissue can contribute to the progression of these diseases by disrupting mitochondrial function, impairing protein kinase C translocation, triggering inflammatory responses, increasing oxidative stress, or causing metabolic disorders. It has been shown that Mg supplementation can improve liver function in some of these liver diseases [50]. Decreased serum Mg is also associated with biopsy-proven hepatic steatosis and steatohepatitis in non-diabetic individuals, caused by insulin resistance [54]. Magnesium can directly affect kidney structure because the kidneys play a vital role in regulating Mg balance. Magnesium is filtered in the glomerulus and reabsorbed in the proximal tubule via a paracellular pathway in the loop of Henle [55]. Although there are fewer animal studies directly examining microscopic structural changes in the kidney due to Mg deficiency, data indicate that Mg supplementation can reduce histopathological changes. A study in mice showed that low dietary Mg intake worsened tubular damage and interstitial fibrosis induced by high phosphate [56]. Some authors suggest that Mg may act by reducing inflammation, oxidative stress and apoptosis, as well as regulating certain signaling pathways associated with kidney fibrosis [57]. Claudin-19 (CLDN-19) is a protein that, along with claudin-16, is expressed in the renal tubules, particularly in regions where Mg and other ions are reabsorbed, including the thick ascending limb (TAL) of the loop of Henle and the distal tubules (DT) [58]. Claudin-19 is localized in the cortical and medullary parts of the TAL, i.e., in the cortex and outer stripe of the outer medulla. In the kidney, CLDN-19 is essential for the paracellular reabsorption of Mg^2+^ and Ca^2+^. Loss of function can result in urinary loss of Mg and Ca [59]. Claudin-19 is encoded by the CLDN-19 gene, and its mutation is associated with an autosomal recessive disorder known as familial hypomagnesemia with hypercalciuria and nephrocalcinosis (FHHNC), characterized by severe loss of Mg and Ca, progressive renal damage and severe ocular abnormalities. Results of our study show that there are no changes in CLDN-19 among the investigated groups of animals, suggesting normal kidney function in the maintenance of Mg homeostasis.

## 4. Materials and Methods

### 4.1. Animals and Treatment

In our experiment, healthy, 60-day-old male Wistar albino (Ws Igs) rats weighing 265–335 g were used (Charles River Laboratories International Inc., Wilmington, MA, USA). The animals were bred and housed in the Unit for Experimental Animals at the Institute for Biological Research “Siniša Stanković”—National Institute of the Republic of Serbia, University of Belgrade, Serbia. The study protocol was approved by the Ethics Committee for the Use of Laboratory Animals of the Institute for Biological Research “Siniša Stanković”—National Institute of the Republic of Serbia, University of Belgrade, Serbia, and by the Department of Animal Welfare and Veterinary Activity, Veterinary Directorate, Ministry of Agriculture, Forestry and Water Management of the Republic of Serbia, License No. 0024321182024/August 27, 2024. The rats were divided into a control group (C) and four experimental groups treated with four different Mg supplements: Mg BT™ (36.4% Mg), Mg Oxi (60.3% Mg), Mg Cit (11.4% Mg) and Mg Gly (14.1% Mg). Each experimental group consisted of six animals, with three housed per cage. All had free access to food (IG–Z–00117, Gebi d.o.o., Čantavir, Serbia), which provided 1405 kJ (333 kcal)/100 g of energy, and water at a constant room temperature of 22 °C and a 12 h light/12 h dark cycle (the chemical composition of standard rat food is provided in the Supplementary Materials, Table S6). The body weight of each animal was measured daily, and the amount of supplements was adjusted so that all rats received 50 mg/kg of elemental Mg per day dissolved in distilled water [60] by gastric gavage during 30 days. Mg BT™ was provided by Open Burch University (Sarajevo, Bosnia and Herzegovina), while all other Mg preparations were Sigma Aldrich products (Saint Louis, MO, USA). The control animals received the same volume of pure distilled water daily by gastric gavage for 30 days to simulate the same experimental conditions as the treated animals. On the 30th day, the animals were sacrificed by decapitation using a Harvard guillotine under isoflurane anesthesia.

### 4.2. Sample Collection

Blood samples without an anticoagulant were collected during euthanasia. After collection, the blood was left to clot at room temperature for 15–30 min, then centrifuged at 1000–2000× *g* for 10 min in a refrigerated centrifuge to remove the clot. The resulting serum was used for biochemical analyses.

Urine was collected after the spontaneous urination of rats on the day of sacrifice. Feces were collected after spontaneous defecation during handling before decapitation or taken with a pinch from the rectum.

Samples of cerebrospinal fluid (CSF) were taken from the animals before euthanasia. CSF collection was performed according to the method of Nirogi et al. (2009) [61] by puncturing the cisterna magna under isoflurane anesthesia. The colorless CSF samples were slowly drawn into the syringe to avoid possible blood contamination.

After decapitation, the liver, kidneys and brain were isolated for further analysis. The cerebral cortex, hypothalamus and hippocampus were isolated from the whole brain. All samples were stored at −80 °C until analysis.

### 4.3. Determination of Mg Concentration

The magnesium concentration in serum, urine and cerebrospinal fluid was determined using a method based on the reaction of Mg from the sample with xylidyl blue in an alkaline medium, resulting in a colored complex measurable spectrophotometrically [62]. Ethyleneglycol-bis(β-aminoethyl)-N,N,Nʹ,Nʹ-tetraacetic acid (EGTA) was added to the reaction mixture to reduce interferences. The prepared sample was measured at 504 nm using a BS-240 Vet Auto Chemistry Analyzer (Mindray, Nanjing, Jiangsu, China).

Brain and feces Mg content was quantified using Abcam’s Magnesium Assay Kit (ab102506), according to the manufacturer’s instructions (Abcam, Cambridge, UK). The assay is based on the specific requirement of glycerol kinase for Mg, in which an enzyme-linked reaction produces an intensely colored compound proportional to the Mg concentration [63]. Brain tissue (cerebral cortex, hypothalamus and hippocampus) was homogenized in four volumes of the Magnesium Assay Buffer supplied with the kit. After centrifugation, 20 µL of the supernatant from each sample was transferred into 96-well plates and incubated with the Magnesium Reaction Mix. Absorbance was measured at 450 nm using an AC-SYNERGY H1 multiplate reader (BioTek, Agilent, Santa Clara, CA, USA) for all samples and standards at 10 min intervals, with four consecutive readings taken to ensure values remained within the linear range. Magnesium concentrations were calculated from the standard curve and expressed as nmol/µL.

### 4.4. Determination of Selected Biochemical Parameters in the Serum

Serum biochemical parameters (albumin, alkaline phosphatase, alanine aminotransferase, aspartate aminotransferase, creatine kinase, cholesterol, creatinine, glucose, total protein, triglycerides and urea) were determined using the BS-240 Vet Auto Chemistry Analyzer (Nanjing, Jiangsu, China), according to standard analytical methods for veterinary analysis (BioSystems S.A., Costa Brava, Barcelona, Spain, www.biosystems.global). Serum sodium (Na), potassium (K) and chloride (Cl) levels were determined using the Diestro Electrolyte Analyzer (Diestro, JC Medicina Electrónica, Buenos Aires, Argentina), according to the manufacturer’s recommendations.

### 4.5. Oral Glucose Tolerance Test—OGTT

The glucose tolerance of rats was assessed using the oral glucose tolerance test (OGTT). This test measures the clearance of orally administered glucose from the blood and is widely used as a diagnostic tool for impaired glucose tolerance in both clinical settings and animal experiments. The OGTT was performed seven days before the end of treatment, following overnight fasting, and began when the first blood sample was taken from a cut at the tip of the tail (time 0) for glucose measurement using a glucometer (Accu-Chek Active, India). Subsequently, a 12.5% glucose solution (2 g/kg body weight) was administered to all groups by oral gavage. Blood glucose levels were then recorded at 30, 60, 90, 120 and 180 min after glucose administration to assess the ability of different Mg supplements to influence glucose clearance from the blood.

The area under the curve (AUC) represents the total increase in blood glucose during the OGTT. AUC was calculated from the OGTT results using the trapezoidal method as follows: a graph was plotted with time (minutes) on the horizontal axis and glucose level (mmol/L) on the vertical axis, and the area under the line connecting the five measured values was calculated by multiplying the time by the glucose level.

### 4.6. Western Blot Analysis

The cortical samples used for RT-PCR and Western blot analyses were obtained from the prefrontal cortex. This region was selected a priori because of its well-established involvement in synaptic plasticity and cognitive processes, as well as evidence that Mg modulates neuronal plasticity-related signaling pathways in the prefrontal cortex [64]. Magnesium is a critical regulator of glutamatergic neurotransmission and synaptic plasticity through its interaction with NMDA receptors. Because cortical circuits are highly dependent on NMDA receptor signaling and neurotrophic support, NR2B and BDNF expression in the cerebral cortex were selected as markers of potential neurobiological effects of Mg supplementation. Cortical brain tissue was homogenized and sonicated in 10 volumes of RIPA buffer (50 mmol Tris–HCl, pH 7.5; 150 mmol NaCl; 1% NP-40; 0.5% Triton X-100; 0.1% SDS; 1 mmol EDTA; 1 mmol EGTA) supplemented with a protease and phosphatase inhibitor cocktail (Roche, Mannheim, Germany). Homogenates were centrifuged at 16,000× *g* for 15 min at 4 °C, and protein concentrations in the supernatants were determined using the Micro BCA Protein Assay Kit (Pierce Biotechnology, Waltham, MA, USA). Equal amounts of protein (20 µg per lane) were separated by 10% SDS-PAGE, transferred onto nitrocellulose membranes (Amersham Bioscience, Little Chalfont, Buckinghamshire, England, UK) and blocked for 1 h at room temperature in either 5% non-fat dry milk (Santa Cruz, Dallas, TX, USA) or 3% BSA (Sigma-Aldrich) in TBST buffer (50 mmol Tris, pH 7.0; 150 mmol NaCl; 0.05% Tween-20). Membranes were incubated overnight at 4 °C with primary antibodies against synaptophysin (SYPH, ab32127), PSD-95 (ab18258), phosphorylated PSD-95 (pSer295, ab76108), and drebrin (ab60933) (all from Abcam) and then for 1 h at room temperature with HRP-conjugated secondary antibodies (bovine anti-rabbit, 1:5000; Santa Cruz, sc-2054). Protein bands were visualized using enhanced chemiluminescence on an iBright imaging system (Thermo Fisher Scientific, Waltham, MA, USA) and quantified with ImageQuant software (Version 5.2, GE Healthcare, Chicago, IL, USA). Target protein intensities were normalized to total protein loading based on Ponceau S staining. For inter-membrane comparisons, a pooled protein sample was used as an internal reference. Protein levels in treated groups were expressed as fold change relative to age-matched controls.

### 4.7. RNA Isolation and Quantitative Real-Time PCR (qRT-PCR)

Total RNA was extracted from cortical brain tissue using the RNeasy Mini Kit (Qiagen, Hilden, Germany), according to the manufacturer’s instructions. RNA concentration and purity were determined spectrophotometrically using a NanoDrop spectrophotometer (Thermo Fisher Scientific). One microgram of total RNA was reverse transcribed into cDNA using the High-Capacity cDNA Reverse Transcription Kit (Applied Biosystems, Foster City, CA, USA), following the manufacturer’s protocol. Primers for target genes were as follows: BDNF IX, F 5′-CACTCCGACCCCGCCCGCCG-3′ and R 5′-TCCACTATCTTCCCCTTTTA-3′; NR2B, F 5′-AATGGCGGATAAGGATGAGT-3′ and R 5′-GGGAAGTAGGTGGTGACGAT-3′; β-actin, F 5′-TGGACATCCGCAAAGACCTGTAC-3′ and R 5′-TCAGGAGGAGCAATGATCTTGA-3′. Quantitative real-time PCR was performed using SYBR Green Master Mix on an Applied Biosystems QuantStudio 3 system. Each reaction was run in triplicate. Gene expression was normalized to β-actin as a housekeeping gene, and relative mRNA levels were calculated using the 2^–ΔΔCt method.

### 4.8. Behavioral Testing

#### 4.8.1. Open Field Test

Locomotor activity and anxiety-like behavior were assessed using the open field test (OF) in Opto-Varimex cages (44.2 × 43.2 × 20 cm; Columbus Instruments, Columbus, OH, USA). Activity was recorded with AutoTrack software (v5.05.09), which sampled movement every 0.1 s and divided the arena into 16 virtual squares (4 × 4 grid). Rats were individually placed in the center of the arena and allowed to explore for 10 min. Behavioral parameters included total distance traveled, ambulatory time, velocity, rearing, grooming, time spent in the central zone, number of center entries, and number of fecal boli.

#### 4.8.2. Light–Dark Box Test

The light–dark box (LDB) test was used to assess anxiety-like behavior in Wistar rats. The test relies on the natural conflict between the rodent’s innate drive to explore novel environments and its aversion to brightly lit, open spaces. The apparatus consisted of two compartments made of opaque Plexiglas: a light chamber (31 × 31 × 36 cm) and a dark chamber (20 × 31 × 36 cm), connected by a 10 × 10 cm opening in the dividing wall. Each animal was placed in the center of the light compartment at the start of the trial, and behavior was recorded for 10 min using a video camera. The following parameters were analyzed: latency to enter the dark chamber, latency to re-enter the light chamber, number of transitions between chambers, and total time spent in the light chamber. Increased time spent in the light compartment and shorter latency to exit the dark compartment was interpreted as reduced anxiety-like behavior.

### 4.9. Histochemistry, Immunofluorescence, and Microscopy of Liver and Kidneys

After removal of the liver and kidneys and recording their respective masses, a macroscopic evaluation of the organs’ morphological characteristics was conducted. For histological analysis and light microscopy, a section of the left median lobe of the liver was excised, while the kidneys were bisected longitudinally to enable more efficient fixation. The tissues were fixed in 10% neutral buffered formalin for 48 h, dehydrated in a series of increasing concentrations of ethanol (50–100%), enlightened in xylol and embedded in Histowax^®^ (Histolab Product AB, Göteborg, Sweden). Serial 5 μm thick tissue sections, obtained using a rotary microtome (HistoCore Biocat, Leica Microsystems, Wetzlar, Germany), were subjected to various histochemical staining procedures.

Hematoxylin and eosin were used as a basic histological stain for assessing cellular morphology and tissue architecture, while Mason’s trichrome staining provided detailed insight into the organization of connective tissue, particularly the distribution of collagen types I and III as described in [65]. Additionally, Periodic Acid–Schiff (PAS) staining and reticulin staining were performed to highlight glycogen and glycoprotein distribution in the organs and to visualize reticulin fibers, respectively [66]. Claudin-19 immunofluorescence was performed in the kidneys after 48 h of LED exposure to reduce autofluorescence. Sections were deparaffinized, rehydrated, antigen-retrieved in citrate buffer (pH 6.0), and permeabilized with 5% Triton X-100. After blocking with normal donkey serum, slides were incubated overnight at 4 °C with anti-Claudin-19 antibody (Invitrogen (Carlsbad, CA, USA)/Thermo Fisher, 1:50), then for 1 h with Alexa Fluor 488 donkey anti-rabbit secondary antibody (1:200) and counterstained with Sytox Orange. Mounting was performed in Mowiol for fluorescence imaging.

Image acquisition and histological analysis of the liver were performed using a microscope (Olympus BX-51, Olympus, Tokyo, Japan) equipped with a CCD video camera (PixeLink, Gloucester, ON, Canada) and image acquisition and analysis software (Visiopharm Integrator System (VIS), ver. 2020.01.3.7887; Visiopharm, Hørsholm, Denmark). The kidney was imaged and histologically analyzed using a Leica DM4B microscope equipped with a Leica DFC 7000T camera. Immunofluorescence images of kidneys were acquired with a Leica TCS SP5 II confocal microscope. For excitation of claudin-19 and Sytox Orange-stained nuclei, argon (488 nm) and helium–neon (543 nm) lasers, respectively, were used.

### 4.10. Statistical Analysis

The results are expressed as the mean ± standard error (SE) of the mean, with the standard deviation (SD) shown in brackets. All data were tested for normality using the Kolmogorov–Smirnov test (n < 50) and for homoscedasticity using Levene’s test. General linear model (GLM) analyses were performed to evaluate treatment effects while controlling for baseline body weight as a potential confounding factor. Initial body weight was included as a covariate to assess whether intergroup differences in body mass contributed to the observed outcomes. Associations between variables were examined using Spearman’s rank correlation coefficient, as this non-parametric method does not require normally distributed data and is suitable for detecting monotonic relationships. For data with a normal distribution, one-way ANOVA followed by the Tukey HSD post hoc test was used to identify differences between groups. For data that did not have a normal distribution, Kruskal–Wallis ANOVA and Dunn’s post hoc test were used. Experimental data for OGTT were analyzed using Dunnett’s multiple comparison test, and for Area Under the Curve (AUC) results, Fisher’s LSD test was used. Different letters or asterisks indicate significant differences between means. Differences were considered statistically significant at *p* < 0.05 with 95% confidence intervals. Data were analyzed using GraphPad Prism for Windows (v.8, San Diego, CA, USA) and STATISTICA software (v.12.5, Palo Alto, CA, USA).

## 5. Conclusions

The present study suggests that administration of the multi-form magnesium supplement Mg BT™ increases serum Mg levels while maintaining glucose homeostasis in healthy Wistar rats under the experimental conditions applied. Changes in cortical synaptic protein markers, including phospho-PSD-95, were observed in Mg BT™-treated animals. However, similar or comparable effects were also detected in groups receiving individual Mg compounds. Behavioral analysis showed preserved locomotor activity with mild anxiolytic-like effects across Mg-treated groups. No histological alterations were observed in liver or kidney tissue, while renal CLDN-19 expression remained unchanged in all experimental groups. These findings indicate that supplementation with Mg BT™ resulted in biological responses across systemic, behavioral and molecular parameters that were comparable to those observed following supplementation with individual magnesium compounds under the conditions of this study. Overall, Mg BT™ was well tolerated and did not induce adverse effects across systemic, behavioral or histological parameters in this model.

## Figures and Tables

**Table 1:** Magnesium concentration (mmol/L) in the serum, urine and feces of animals at the start and at the end of the experiment.

Mg	Serum		Urine		Feces	
(mmol/L)	Start	End	Start	End	Start	End
Control	0.82 ± 0.10 (0.23)	0.79 ± 0.02 (0.04)	1.39 ± 0.01 (0.02)	1.34 ± 0.02 (0.05)	16.10 ± 1.17 (2.33)	17.87 ± 4.60 (10.29)
Mg BT™	0.74 ± 0.02 (0.05)	0.93 ± 0.01 ^S,C^ (0.03)	1.35 ± 0.02 (0.04)	1.32 ± 0.03 (0.07)	15.29 ± 1.24 (2.77)	21.70 ± 4.05 ^S^ (9.91)
Mg Oxi	0.70 ± 0.04 (0.10)	0.89 ± 0.03 ^S^ (0.07)	1.32 ± 0.05 (0.12)	1.26 ± 0.03 ^S^ (0.05)	17.41 ± 0.97 (1.93)	14.12 ± 1.70 (4.16)
Mg Cit	0.82 ± 0.07 (0.18)	0.85 ± 0.03 (0.07)	1.44 ± 0.04 (0.09)	1.20 ± 0.03 ^S,C^ (0.06)	15.13 ± 2.74 (6.12)	19.98 ± 5.18 (12.68)
Mg Gly	0.75 ± 0.05 (0.17)	0.83 ± 0.02 (0.09)	1.38 ± 0.01 (0.02)	1.16 ± 0.09 ^S,C^ (0.21)	16.98 ± 2.52 (6.17)	21.37 ± 3.09 (7.58)
	F = 2.857, df = 57 (9,48), *p* = 0.0088		F = 4.251, df = 50 (9,41), *p* = 0.0006		F = 1.382, df = 51 (9,42), *p* = 0.0396	

Data are expressed as Mean ± standard error (SE). Standard deviations (SDs) are presented in brackets. One-way ANOVA followed by Tukey (HSD) post hoc test was performed to seek significant differences between means. Significantly different at *p* < 0.05; ^S^ With respect to start of the experiment; **^C^** Differences with respect to control. Exact *p* values are given in the text.

**Table 2:** Magnesium concentration (mmol/L) in the cerebrospinal fluid (CSF) and in different brain regions at the start and after 30 days of the experiment.

	CSF (mmol/L)	Cerebral Cortex (mmol/L)	Hippocampus (mmol/L)	Hypothalamus (mmol/L)
Control	0.71 ± 0.01 (0.01)	0.52 ± 0.06 (0.028)	0.71 ± 0.08 (0.04)	0.36 ± 0.13 (0.06)
Mg BT™	0.70 ± 0.02 (0.05)	0.53 ± 0.03 (0.015)	0.72 ± 0.15 (0.06)	0.38 ± 0.12 (0.05)
Mg Oxi	0.68 ± 0.01 (0.02)	0.52 ± 0.09 (0.04)	0.70 ± 0.14 (0.06)	0.39 ± 0.078 (0.03)
Mg Cit	0.67 ± 0.01 (0.03)	0.48 ± 0.11 (0.04)	0.70 ± 0.06 (0.03)	0.33 ± 0.11 (0.04)
Mg Gly	0.67 ± 0.01 (0.03)	0.60 ± 0.11 (0.05)	0.79 ± 0.10 (0.042)	0.43 ± 0.086 (0.04)
	F = 0.6923, df = 31 (5,26) *p* = 0.6338	F = 1.1481, df = 28 (4,24) *p* = 0.2391	F = 0.9776, df = 34 (5,29) *p* = 0.4480	F = 0.2161, df = 34 (5,29) *p* = 0.0862

Data are expressed as Mean ± standard error (SE). Standard deviations (SDs) are presented in brackets. One-way ANOVA followed by Tukey (HSD) post hoc test was performed to seek significant differences between means. Exact *p* values are given in the text.

**Table 3:** Oral glucose tolerance test in rats after treatment with Mg supplements.

	Blood Glucose Level (mmol/L)				
	0 min	30 min	60 min	120 min	180 min
Control	5.32 ± 0.17 (0.38)	8.40 ± 0.36 **** (0.81)	8.18 ± 0.57 **** (1.27)	5.92 ± 0.09 (0.22)	5.76 ± 0.10 (0.23)
Mg5 BT™	4.30 ± 0.06 (0.14)	8.17 ± 0.26 **** (0.65)	8.67 ± 0.23 **** (0.56)	5.70 ± 0.25 **** (0.61)	4.86 ± 0.09 (0.23)
Mg Oxi	5.10 ± 0.23 (0.57)	7.32 ± 0.24 **** (0.59)	7.25 ± 0.28 **** (0.69)	5.88 ± 0.36 (0.87)	5.35 ± 0.18 (0.45)
Mg Cit	4.35 ± 0.15 (0.36)	9.01 ± 0.43 **** (1.06)	8.37 ± 0.36 **** (0.89)	5.78 ± 0.27 ** (0.66)	4.98 ± 0.22 (0.53)
Mg Gly	4.82 ± 0.16 (0.39)	9.22 ± 0.39 **** (0.96)	9.28 ± 0.66 **** (1.61)	6.90 ± 0.52 ** (1.27)	5.40 ± 0.30 (0.74)

Data are expressed as Mean ± standard error (SE). Standard deviations (SDs) are presented in brackets. One-way ANOVA followed by Dunnett’s multiple comparison test was performed to seek significant differences between means. **** *p* < 0.0001; ** *p* < 0.01 statistically significant compared with the mean at 0 min in each group. ANOVA effects and exact *p* values are given in Supplementary Material (Table S5).

**Table 4:** Changes in the cortical expression of synaptophysin (SYPH), PSD-95, phospho-PSD-95, phospho-PSD-95/PSD-95 and drebrin levels, of animals treated with different Mg supplements. The data are expressed as relative values of protein expression relative to the respective controls.

	SYPH	PSD-95	p-PSD-95	p-PSD-95/PSD-95	Drebrin
Control	1.00 ± 0.04 (0.90)	1.00 ± 0.05 (0.11)	1.99 ± 0.04 (0.098)	1.01 ± 0.08 (0.18)	1.00 ± 0.04 (0.08)
Mg BT™	0.76 ± 0.03 ^C^ (0.06)	1.03 ± 0.03 (0.08)	1.01 ± 0.04 (0.10)	0.99 ± 0.03 (0.08)	1.09 ± 0.08 (0.19)
Mg Oxi	0.95 ± 0.03 ^BT™^ (0.07)	0.91 ± 0.04 (0.09)	1.28 ± 0.09 ^C^ (0.22)	1.41 ± 0.09 ^C^ (0.22)	1.38 ± 0.22 (0.55)
Mg Cit	1.04 ± 0.03 ^BT™^ (0.07)	1.09 ± 0.06 (0.16)	1.49 ± 0.11 (0.28)	1.39 ± 0.13 (0.32)	1.96 ± 0.28 ^C^ (0.69)
Mg Gly	0.97 ± 0.02 ^BT™^ (0.05)	1.07 ± 0.04 (0.09)	1.39 ± 0.15 (0.37)	1.30 ± 0.13 (0.33)	1.89 ± 0.21 ^C^ (0.52)
	F = 12.65, df = 28 (4,24) *p* < 0.0001	F = 2.519, df = 28 (4,24) *p* = 0.0678	F = 3.745, df = 23 (3,20) *p* = 0.0276	F = 4.018, df = 23 (3,20) *p* = 0.0124	F = 4.919, df = 28 (4,24) *p* = 0.0049

Data are expressed as Mean ± standard error (SE). Standard deviations (SDs) are presented in brackets. One-way ANOVA followed by Tukey (HSD) post hoc test was performed to seek significant differences between means. Significantly different at *p* < 0.05. ^C^ Differences with respect to control; ^BT™^ Differences with respect to Mg BT™. Exact *p* values are given in the text.

## Data Availability

The datasets used and analyzed during the current study are available from the corresponding author on reasonable request.

## Supplementary Materials

The following supporting information can be downloaded at: https://www.mdpi.com/article/10.3390/ijms27156581/s1.

## Abbreviations

The following abbreviations are used in this manuscript:

Mg BT™	Magnesium Breakthrough
Mg Oxi	Magnesium Oxide
Mg Cit	Magnesium Citrate
Mg Gly	Magnesium Glycinate
RDA	Recommended Dietary Allowance
CSF	Cerebrospinal Fluid
OGTT	Oral Glucose Tolerance Test
AUC	Area Under the Curve
LDB	Light–Dark Box
SYPH	Synaptophysin
PSD95	Postsynaptic Density Protein95
BDNF	Brain-Derived Neurotrophic Factor
CLDN-19	Claudine-19
TAL	Thick Ascending Limb of Henle’s
