# Supplementary material for: Integrated Systemic and Neurobiological Effects of a Multi-Form Magnesium Supplement Compared to Single Magnesium Forms in Rats
Source: Int J Mol Sci. 2026 Jul 24;27(15):6581. doi: 10.3390/ijms27156581 (PMC13465013; doi:10.3390/ijms27156581)
Supplement: Supplementary file 1 [file ijms-27-06581-s001.zip › Supplementary_Table S1.pdf]

Integrated Systemic and Neurobiological Effects of the Multi-Form Magnesium Supplement Magnesium Breakthrough in Rats Compared to Single Magnesium Forms

Muamer Dizdar [1], Monia Obučić [2], Neira Crnčević [2], Svetlana Dinić [3], Aleksandra Uskoković [3], Jelena Arambašić Jovanović [3], Slavica Borković-Mitić [3], Aleksandra Mladenović [3], Desanka Milanović [3], Smilja Praćer [3], Nataša Nestorović [3], Milica Manojlović-Stojanoski [3], Mirjana Mihailović [3] and Slađan Pavlović [3,*]

[1] Faculty of Science, University of Sarajevo, Zmaja od Bosne 33-35, 71000 Sarajevo, Bosnia and Herzegovina;
muamer.dizdar@pmf.unsa.ba (M.D.)
[2] Faculty of Engineering, Natural and Medical Sciences, International Burch University, Francuske Revolucije bb, 71000 Sarajevo, Bosnia and Herzegovina; monia.avdic@ibu.edu.ba (M.O.); neira.crncevic@ibu.edu.ba (N.C)
[3] Institute for Biological Research "Siniša Stanković" – National Institute of the Republic of Serbia, University of Belgrade, Bulevar despota Stefana 142, 11108 Belgrade, Serbia; sdinic@ibiss.bg.ac.rs (S.D.); auskokovic@ibiss.bg.ac.rs (A.U.); jelena.arambasic@ibiss.bg.ac.rs (J.A.J.); borkos@ibiss.bg.ac.rs (S.B.-M.); anamikos@ibiss.bg.ac.rs (A.M.); desan@ibiss.bg.asc.rs (D.M.); smilja.pracer@ibiss.bg.ac.rs (S.P.); rnata@ibiss.bg.ac.rs (N.N.) manojlo@ibiss.bg.ac.rs (M.M.-S.); mirjanam@ibiss.bg.ac.rs (M.M.); sladjan@ibiss.bg.ac.rs (S.Z.P.)
* Correspodence: sladjan@ibiss.bg.ac.rs (S.Z.P.);

Supplementary material

Table S1. A) General linear model (GLM) between baseline body weight as a potential confounding factor and selected analyses. B) Spearmman rank correlations between baseline body weight and selected analyses.

A) General Linear Model (GLM)

Body mass gain (g), absolute (LW) and relative liver weight (RLW), absolute (LKW) and relative left kidney weight (relative LKW) and absolute (RKW) and relative right kidney weight (relative RKW):

	Multivariate Tests of Significance. Sigma-restricted parameterization Effective hypothesis decomposition					
Effect	Test	Value	F	Effect df	Error df	p
Intercept	Wilks	0.482881	2.498776	6	14.00000	0.074119
**Groups**	Wilks	0.197359	1.235114	24	50.05021	0.259336
Mass beginning	Wilks	0.493179	2.397873	6	14.00000	0.083311
**Groups***Mass beginning	Wilks	0.176920	1.340821	24	50.05021	0.188422

Serum biochemical parameters:

	Multivariate Tests of Significance. Sigma-restricted parameterization Effective hypothesis decomposition					
Effect	Test	Value	F	Effect df	Error df	p
Intercept	Wilks	0.004728	35.08503	6	1.000000	0.128519
**Groups**	Wilks	0.000439	1.59940	24	4.698624	0.327060
Mass beginning	Wilks	0.015792	10.38714	6	1.000000	0.233156
**Groups***Mass beginning	Wilks	0.000379	1.67674	24	4.698624	0.306496

Mg concentrations in the serum, urine, feces, cerebral cortex, hippocampus, hypothalamus and CSF:

	Multivariate Tests of Significance. Sigma-restricted parameterization Effective hypothesis decomposition					
Effect	Test	Value	F	Effect df	Error df	p
Intercept	Wilks	0.298636	2.348555	4	4.00000	0.214284
**Groups**	Wilks	0.052021	1.311218	16	12.85783	0.315557
Mass beginning	Wilks	0.363694	1.749567	4	4.00000	0.300605
**Groups***Mass beginning	Wilks	0.050628	1.330092	16	12.85783	0.306326

Synaptophysin, PSD95, Phospho-PSD95, drebrin, NR2B, BDNF:

	Multivariate Tests of Significance. Sigma-restricted parameterization Effective hypothesis decomposition					
Effect	Test	Value	F	Effect df	Error df	p
Intercept	Wilks	0.837192	0.421348	6	13.00000	0.851973
**Groups**	Wilks	0.185851	1.202686	24	46.56162	0.288320
Mass beginning	Wilks	0.632709	1.257761	6	13.00000	0.340659
**Groups***Mass beginning	Wilks	0.194216	1.163272	24	46.56162	0.321431

Blood glucose levels:

	Multivariate Tests of Significance. Sigma-restricted parameterization Effective hypothesis decomposition					
Effect	Test	Value	F	Effect df	Error df	p
Intercept	Wilks	0.813436	0.688059	5	15.00000	0.639978
**Groups**	Wilks	0.290043	1.146711	20	50.69925	0.336671
Mass beginning	Wilks	0.825549	0.633945	5	15.00000	0.677122
**Groups***Mass beginning	Wilks	0.260317	1.268717	20	50.69925	0.242863

Behaviour: distance traveled, ambulatory time, exploratory time, stereotypic activity, velocity, OF entrance center, OF entrance perifery, time spent center, time spent perifery, latency before entering dark chamber, time until first exit to light chamber, time spent in light chamber, number of entries into light chamber:

	Multivariate Tests of Significance. Sigma-restricted parameterization Effective hypothesis decomposition					
Effect	Test	Value	F	Effect df	Error df	p
Intercept	Wilks	0.048182	3.039195	13	2.000000	0.274560
**Groups**	Wilks	0.000056	2.188873	52	9.856850	0.091914
Mass beginning	Wilks	0.046964	3.122009	13	2.000000	0.268505
**Groups***Mass beginning	Wilks	0.000049	2.271955	52	9.856850	0.082088

B) Spearmman correlations

Body mass gain (g), Absolute (LW) and relative liver weight (RLW), absolute (LKW) and relative left kidney weight (relative RLKW) and absolute (RKW) and relative right kidney weight (relative RRKW):

Pair of Variables	Spearman Rank Order Correlations pairwise deleted Marked correlations are significant at p <.05000			
	Valid N	Spearman R	t(N-2)	p-value
Mass at beginning (g) & **Body mass gain (g)**	29	0.000493	0.00256	0.997974
Mass at beginning & **LW**	29	0.197339	1.045974	0.304847
Mass at beginning & **RLW**	29	0.073901	0.385052	0.703215
Mass at beginning **& LKW**	29	0.247873	1.329475	0.194816
Mass at beginning & **RLKW**	29	0.175391	0.925708	0.362798
Mass at beginning & **RKW**	29	0.310574	1.697747	0.101054
Mass at beginning & **RRKW**	29	0.103168	0.538951	0.594337

Serum biochemical parameters:

Pair of Variables	Spearman Rank Order Correlations (STATISTIKA SERUM BIOHEMIJA-GLM) MD pairwise deleted Marked correlations are significant at p <.05000			
	Valid N	Spearman R	t(N-2)	p-value
Mass beginning & **Albumin (g/L)**	29	-0.324424	-1.78215	0.085975
Mass beginning & **ALP (U/L)**	27	-0.138931	-0.70146	0.489495
Mass beginning & **ALT (U/L)**	28	-0.264404	-1.39795	0.173941
Mass beginning & **AST (U/L)**	24	-0.137856	-0.65284	0.520627
Mass beginning & **Ck (U/L)**	26	0.072491	0.35607	0.724900
Mass beginning & **Chol (mmol/L)**	29	-0.222729	-1.18715	0.245507
Mass beginning & **Crea (mmol/L)**	26	-0.070546	-0.34647	0.732010
Mass beginning & **Glu (mmol/L)**	24	-0.342920	-3.93711	0.070335
Mass beginning & **Prot-T (g/L)**	24	-0.250989	-3.09686	0.052644
Mass beginning & **Trig (mmol/L)**	24	-0.238416	-1.15148	0.261897
Mass beginning & **Urea (mmol/L)**	22	0.077599	0.34808	0.731416
Mass beginning & **Ca (mmol/L)**	29	-0.000863	-0.00448	0.996455
Mass beginning & **Na (mmol/L)**	28	-0.068866	-0.35199	0.727685
Mass beginning & **K (mmol/L)**	27	0.244885	1.26288	0.218285
Mass beginning & **Cl (mmol/L)**	28	0.167944	0.86869	0.392966

Mg concentrations in the serum, urine, feces, cerebral cortex, hippocampus, hypothalamus and CSF:

Pair of Variables	Spearman Rank Order Correlations pairwise deleted Marked correlations are significant at p <.05000			
	Valid N	Spearman R	t(N-2)	p-value
Mass beginning & **Mg Serum**	29	-0.119403	-0.624907	0.537278
Mass beginning & **Mg Urin**	22	0.279740	1.303060	0.207358
Mass beginning & **Mg Feces**	24	0.220870	1.062203	0.299665
Mass beginning & **Mg CSF**	29	0.182854	0.966431	0.342406
Mass beginning & **CTX**	29	-0.186483	-0.986297	0.332744
Mass beginning & **Hipp**	29	-0.001357	-0.007049	0.994428
Mass beginning & **Hypo**	29	-0.186474	-0.986245	0.332769

Synaptophysin, PSD95, Phospho-PSD95, drebrin, NR2B, BDNF:

Pair of Variables	Spearman Rank Order Correlations pairwise deleted Marked correlations are significant at p <.05000			
	Valid N	Spearman R	t(N-2)	p-value
Mass beginning & **SPF**	29	0.215544	1.14696	0.261461
Mass beginning & **PSDp5**	29	0.172189	0.90828	0.371764
Mass beginning & **PhosphoPSD95**	29	-0.250523	-1.34464	0.189930
Mass beginning & **Drebrin**	29	-0.263087	-1.41695	0.167932
Mass beginning & **NR2B**	29	0.212834	1.13185	0.267650
Mass beginning & **BDNF**	28	0.368702	2.02251	0.053523

Blood glucose levels:

Pair of Variables	Spearman Rank Order Correlations pairwise deleted Marked correlations are significant at p <.05000			
	Valid N	Spearman R	t(N-2)	p-value
Mass beginning & **0 min**	29	0.309383	1.690546	0.102438
Mass beginning & **30 min**	29	-0.102852	-0.537287	0.595470
Mass beginning & **60 min**	29	-0.054507	-0.283650	0.778842
Mass beginning & **120 min**	29	0.160450	0.844667	0.405719
Mass beginning & **160 min**	29	0.357364	1.988209	0.057014

Behaviour: distance traveled, ambulatory time, exploratory time, stereotypic activity, velocity, OF entrance center, OF entrance perifery, time spent center, time spent perifery, latency before entering dark chamber, time until first exit to light chamber, time spent in light chamber, number of entries into light chamber:

Pair of Variables	Spearman Rank Order Correlations pairwise deleted Marked correlations are significant at p <.05000			
	Valid N	Spearman R	t(N-2)	p-value
Mass beginning & **DT**	29	-0.055179	-0.28716	0.776184
Mass beginning & **Amb. time**	29	-0.036211	-0.18828	0.852063
Mass beginning & **Expl. act.**	29	0.033148	0.17234	0.864456
Mass beginning & **Stereo. act.**	28	0.112815	0.57894	0.567611
Mass beginning & **Velocity**	29	-0.129573	-0.67900	0.502915
Mass beginning & **OF entr. cent.**	28	-0.344251	-1.86962	0.072839
Mass beginning & **OF entr. per.**	29	-0.268785	-1.45001	0.158574
Mass beginning & **OF time cent.**	28	-0.361221	-1.97524	0.058946
Mass beginning & **OF time per.**	29	0.137702	0.72240	0.476259
Mass beginning & **Latency**	28	0.197745	1.02862	0.313134
Mass beginning & **Time until**	27	0.218965	1.12205	0.272502
Mass beginning & **Time spent**	28	-0.323584	-1.74378	0.093013
Mass beginning & **No ent. light**	28	-0.320668	-1.72625	0.096169
