# Supplementary material for: Integrated Systemic and Neurobiological Effects of a Multi-Form Magnesium Supplement Compared to Single Magnesium Forms in Rats
Source: Int J Mol Sci. 2026 Jul 24;27(15):6581. doi: 10.3390/ijms27156581 (PMC13465013; doi:10.3390/ijms27156581)
Supplement: Supplementary file 1 [file ijms-27-06581-s001.zip › Supplementary_Table S2.pdf]

Integrated Systemic and Neurobiological Effects of the Multi-Form Magnesium Supplement Magnesium Breakthrough in Rats Compared to Single Magnesium Forms

Muamer Dizdar [1], Monia Obučić [2], Neira Crnčević [2], Svetlana Dinić [3], Aleksandra Uskoković [3], Jelena Arambašić Jovanović [3], Slavica Borković-Mitić [3], Aleksandra Mladenović [3], Desanka Milanović [3], Smilja Praćer [3], Nataša Nestorović [3], Milica Manojlović-Stojanoski [3], Mirjana Mihailović [3] and Slađan Pavlović [3,*]

[1] Faculty of Science, University of Sarajevo, Zmaja od Bosne 33-35, 71000 Sarajevo, Bosnia and Herzegovina;
muamer.dizdar@pmf.unsa.ba (M.D.)
[2] Faculty of Engineering, Natural and Medical Sciences, International Burch University, Francuske Revolucije bb, 71000 Sarajevo, Bosnia and Herzegovina; monia.avdic@ibu.edu.ba (M.O.); neira.crncevic@ibu.edu.ba (N.C)
[3] Institute for Biological Research "Siniša Stanković" – National Institute of the Republic of Serbia, University of Belgrade, Bulevar despota Stefana 142, 11108 Belgrade, Serbia; sdinic@ibiss.bg.ac.rs (S.D.); auskokovic@ibiss.bg.ac.rs (A.U.); jelena.arambasic@ibiss.bg.ac.rs (J.A.J.); borkos@ibiss.bg.ac.rs (S.B.-M.); anamikos@ibiss.bg.ac.rs (A.M.); desan@ibiss.bg.asc.rs (D.M.); smilja.pracer@ibiss.bg.ac.rs (S.P.); rnata@ibiss.bg.ac.rs (N.N.) manojlo@ibiss.bg.ac.rs (M.M.-S.); mirjanam@ibiss.bg.ac.rs (M.M.); sladjan@ibiss.bg.ac.rs (S.Z.P.)
* Correspodence: sladjan@ibiss.bg.ac.rs (S.Z.P.);

Supplementary material

Table S2. Average weights (g) of experimental animals at the beginning of the experiment, at the end of the experiment (g) and body mass gain (g).

Groups	Mass at the beginning of the experiment (g)	Mass at the end of the experiment (g)	Body mass gain (g)
Control	334.20 ± 2.31 (5.16)	417.00 ± 8.46 (18.90)	82.80 ± 7.40 (16.55)
Mg BT™	304.50 ± 17.24 (42.23)	372.17 ± 22.39 (54.83)	67.66 ± 8.12 (19.89)
Mg Oxi	298.33 ± 8.52 (20.88)	347.67 ± 8.33 (20.41)	49.33 ± 4.01^{C} (9.83)
Mg Cit	269.66 ± 15.49 (37.96)	350.33 ± 14.29 (35.00)	80.66 ± 4.21 (10.32)
Mg Gly	295.00 ± 16.78 (41.10)	369.50 ± 14.00 (34.29)	74.50 ± 9.39 (23.00)
	F=2.599, df=28 (4,24) p=0.0616	F=3.217 df=28 (4,24) p=0.03	F=3.748 df=28 (4,24) p=0.0166

Data are expressed as Mean ± standard error (SE). Standard deviations (SD) are presented in the brackets.
One–way ANOVA followed by Tukey (HSD) post hoc test was performed to seek significant differences between means.
Significantly different at $p < 0.05$: ^{C}in respect to control group. Exact p values are given in the text.
