# Supplementary material for: Integrated Systemic and Neurobiological Effects of a Multi-Form Magnesium Supplement Compared to Single Magnesium Forms in Rats
Source: Int J Mol Sci. 2026 Jul 24;27(15):6581. doi: 10.3390/ijms27156581 (PMC13465013; doi:10.3390/ijms27156581)
Supplement: Supplementary file 1 [file ijms-27-06581-s001.zip › Supplementary_Table S3.pdf]

Integrated Systemic and Neurobiological Effects of the Multi-Form Magnesium Supplement Magnesium Breakthrough in Rats Compared to Single Magnesium Forms

Muamer Dizdar [1], Monia Obučić [2], Neira Crnčević [2], Svetlana Dinić [3], Aleksandra Uskoković [3], Jelena Arambašić Jovanović [3], Slavica Borković-Mitić [3], Aleksandra Mladenović [3], Desanka Milanović [3], Smilja Praćer [3], Nataša Nestorović [3], Milica Manojlović-Stojanoski [3], Mirjana Mihailović [3] and Slađan Pavlović [3,*]

[1] Faculty of Science, University of Sarajevo, Zmaja od Bosne 33-35, 71000 Sarajevo, Bosnia and Herzegovina;
muamer.dizdar@pmf.unsa.ba (M.D.)
[2] Faculty of Engineering, Natural and Medical Sciences, International Burch University, Francuske Revolucije bb, 71000 Sarajevo, Bosnia and Herzegovina; monia.avdic@ibu.edu.ba (M.O.); neira.crncevic@ibu.edu.ba (N.C)
[3] Institute for Biological Research "Siniša Stanković" – National Institute of the Republic of Serbia, University of Belgrade, Bulevar despota Stefana 142, 11108 Belgrade, Serbia; sdinic@ibiss.bg.ac.rs (S.D.); auskokovic@ibiss.bg.ac.rs (A.U.); jelena.arambasic@ibiss.bg.ac.rs (J.A.J.); borkos@ibiss.bg.ac.rs (S.B.-M.); anamikos@ibiss.bg.ac.rs (A.M.); desan@ibiss.bg.asc.rs (D.M.); smilja.pracer@ibiss.bg.ac.rs (S.P.); rnata@ibiss.bg.ac.rs (N.N.) manojlo@ibiss.bg.ac.rs (M.M.-S.); mirjanam@ibiss.bg.ac.rs (M.M.); sladjan@ibiss.bg.ac.rs (S.Z.P.)
* Correspodence: sladjan@ibiss.bg.ac.rs (S.Z.P.);

Supplementary material

Table S3. Absolute (LW) and relative liver weight (RLW), absolute (LKW) and relative left kidney weight (relative LKW) and absolute (RKW) and relative right kidney weight (relative RKW) of control and treated rats.

	LW (g)	RLW (%)	LKW (g)	Relative LKW (%)	RKW (g)	Relative RKW (%)
Control	13.94 ± 0.83	3.34 ± 0.12	1.47 ± 0.06	1.51 ± 0.09	0.35 ± 0.01	0.36 ± 0.04
Mg BT™	12.71 ± 2.85	3.39 ± 0.28	1.28 ± 0.21*	1.33 ± 0.20	0.34 ± 0.02	0.36 ± 0.03
Mg Oxi	10.38 ± 0.75	2.99 ± 0.19*	1.13 ± 0.07*	1.20 ± 0.08*	0.33 ± 0.02	0.35 ± 0.02
Mg Cit	11.94 ± 1.42	3.41 ± 0.17	1.19 ± 0.12*	1.16 ± 0.16*	0.34 ± 0.00	0.33 ± 0.02
Mg Gly	13.12 ± 1.63	3.55 ± 0.19	1.31 ± 0.11	1.29 ± 0.13*	0.35 ± 0.02	0.35 ± 0.01
	F=1.568 df=28 (4,24) *p*=0.0503	F=6.496 df=28 (4,24) *p*=0.0011	F=5.773 df=28 (4,24) *p*=0.0021	F=2.994 df=28 (4,24) *p*=0.0388	F=2.256 df=28 (4,24) *p*=0.4882	F=1.445 df=28 (4,24) *p*=0.2498

All values are presented as mean ± SD. * – statistical significance in respect to Control; $p < 0.05$ was considered significant. Exact *p* values are given in the text.
