# Supplementary material for: Integrated Systemic and Neurobiological Effects of a Multi-Form Magnesium Supplement Compared to Single Magnesium Forms in Rats
Source: Int J Mol Sci. 2026 Jul 24;27(15):6581. doi: 10.3390/ijms27156581 (PMC13465013; doi:10.3390/ijms27156581)
Supplement: Supplementary file 1 [file ijms-27-06581-s001.zip › Supplementary_Table S4.pdf]

Integrated biological responses in rats treated with the blend Magnesium Breakthrough™ (Mg BT™) compared to selected magnesium supplements

Muamer Dizdar [1], Monia Obučić [2], Neira Crnčević [2], Svetlana Dinić [3], Aleksandra Uskoković [3], Jelena Arambašić Jovanović [3], Slavica Borković-Mitić [3], Aleksandra Mladenović [3], Desanka Milanović [3], Smilja Praćer [3], Nataša Nestorović [3], Milica Manojlović-Stojanoski [3], Mirjana Mihailović [3] and Slađan Pavlović [3,*]

[1] Faculty of Science, University of Sarajevo, Zmaja od Bosne 33-35, 71000 Sarajevo, Bosnia and Herzegovina; muamer.dizdar@pmf.unsa.ba (M.D.)

[2] Faculty of Engineering, Natural and Medical Sciences, International Burch University, Francuske Revolucije bb, 71000 Sarajevo, Bosnia and Herzegovina; monia.avdic@ibu.edu.ba (M.O.); neira.crncevic@ibu.edu.ba (N.C)

[3] Institute for Biological Research "Siniša Stanković" – National Institute of the Republic of Serbia, University of Belgrade, Bulevar despota Stefana 142, 11108 Belgrade, Serbia; sdinic@ibiss.bg.ac.rs (S.D.); auskokovic@ibiss.bg.ac.rs (A.U.); jelena.arambasic@ibiss.bg.ac.rs (J.A.J.); borkos@ibiss.bg.ac.rs (S.B.-M.); anamikos@ibiss.bg.ac.rs (A.M.); desan@ibiss.bg.asc.rs (D.M.); smilja.pracer@ibiss.bg.ac.rs (S.P.); rnata@ibiss.bg.ac.rs (N.N.) manojlo@ibiss.bg.ac.rs (M.M.-S.); mirjanam@ibiss.bg.ac.rs (M.M.); sladjan@ibiss.bg.ac.rs (S.Z.P.)

* Correspodence: sladjan@ibiss.bg.ac.rs (S.Z.P.);

Supplementary material

Table S4: Selected biochemical analyses in the serum of animals at the end of experiment: albumin (g/L), alkaline phosphatase (ALP, U/L), alanine amino transaminase (ALT, U/L), aspartate amino transaminase (AST, U/L), creatine kinase (U/L), total cholesterol (Chol, mmol/L), creatinine (Crea, µmol/L), glucose (GLU, mmol/L), total protein content (Prot-T, g/L), triglycerides (mmol/L), urea (mmol/L), calcium (Ca, mmol/L), sodium (Na, mmol/L), potassium (K, mmol/L) and chlorides (Cl, mmol/L). Reference values are given in the brackets.

**Serum**	Control	Mg BT™	Mg Oxi	Mg Cit	Mg Gly	ANOVA effects
Albumin	32.16 ± 2.23	60.87 ± 2.37 ^{C}	47.90 ± 2.46	49.17 ± 5.02	47.98 ± 4.52	F=7.446, df=28 (4,24)
(31-46 g/L)	(4.99)	(5.82)	(6.02)	(12.29)	(11.06)	*p*=0.0005
ALP	275.34 ± 44.44	286.26 ± 20.21	246.86 ± 37.07	295.97 ± 22.76	313.82 ± 15.15	F=0.7581, df=26 (4,22)
(95-611 U/L)	(99.37)	(45.19)	(82.89)	(55.76)	(37.16)	*p*=0.5636
ALT	68.42 ± 5.24	113.00 ± 10.36	99.63 ± 15.35	96.90 ± 18.53	117.85 ± 18.08^{C}	F=1.663, df=27 (4,23)
(67-117 U/L)	(11.72)	(25.38)	(37.60)	(41.43)	(44.29)	*p*=0.03228
AST	279.86 ± 41.01	509.02 ± 51.75	648.24 ± 72.35 ^{C}	503.45 ± 72.06	598.97 ± 35.47 ^{C}	F=6.280, df=23 (4,19)
(165-349 U/L)	(91.69)	(126.77)	(161.79)	(144.11)	(70.94)	*p*=0.0021
CK	4275.60 ± 298.22	3253.00 ± 420.39	3839.50 ± 332.12	4157.75 ± 391.14	3583.40 ± 344.69	F=2.183, df=25 (4,21)
(400-4000 U/L)	(666.84)	(1029.74)	(813.53)	(782.29)	(770.74)	*p*=0.1060
Chol	1.89 ± 0.09	2.76 ± 0.16 ^{C}	2.22 ± 0.09	2.37 ± 0.20	2.36 ± 0.19	F=3.750, df=28 (4,24)
(1.5-4.0 mmol/L)	(0.21)	(0.39)	(0.22)	(0.48)	(0.47)	*p*=0.0166
Crea	47.40 ± 1.99	39.40 ± 2.44	43.67 ± 1.78	44.40 ± 4.54	52.80 ± 5.88	F=1.866, df=25 (4,21)
(60-91 µmol/L)	(4.45)	(5.46)	(4.37)	(10.16)	(13.14)	*p*=0.1541
Glu	7.58 ± 0.18	10.48 ± 0.47 ^{C}	7.54 ± 0.49	10.63 ± 0.13^{C}	9.44 ± 0.54	F=11.23, df=23 (4,19)
(5.7-11.2 mmol/L)	(0.40)	(1.16)	(1.09)	(0.23)	(1.21)	*p*=0.0001
Prot-T	66.50 ± 2.99	116.70 ± 3.85 ^{C}	96.24 ± 4.93	80.90 ± 8.52^{MgBT}	88.44 ± 9.27	F=10.82, df=23 (4,19)
(55-71 g/L)	(6.69)	(9.43)	(11.02)	(14.76)	(20.73)	*p*=0.0001
Trig	1.52 ± 0.10	1.85 ± 0.15	1.25 ± 0.29	1.46 ± 0.34	1.42 ± 0.29	F=1.042, df=23 (4,19)
(0.67-3.56 mmol/L)	(0.23)	(0.37)	(0.65)	(0.59)	(0.67)	*p*=0.4115
Urea	7.28 ± 0.33	7.57 ± 0.19	7.45 ± 0.67	7.05 ± 0.25	7.32 ± 0.24	F=0.2169, df=22 (4,18)
(6.1-8.3 mmol/L)	(0.74)	(0.45)	(1.34)	(0.35)	(0.54)	*p*=0.9256
Ca	1.76 ± 0.12	1.96 ± 0.04	1.79 ± 0.04	1.81 ± 0.08	1.90 ± 0.07	F=1.358, df=28 (4,24)
(2.21-2.94 mmol/L)	(0.26)	(0.11)	(0.09)	(0.20)	(0.16)	*p*=0.278
Na	138.68 ± 3.72	127.40 ± 4.43	142.41 ± 3.40	142.12 ± 1.97	140.11 ± 1.99	F=1.358, df=27 (4,23)
(138-155 mmol/L)	(7.43)	(10.86)	(8.33)	(4.82)	(4.96)	*p*=0.091
K	5.08 ± 1.04	6.12 ±1.30	7.91 ± 2.46	4.87 ± 0.16	4.87 ± 0.16	F=1.406, df=26 (4,22)
(4.6-6.mmol/L)	(2.08)	(3.18)	(6.04)	(0.38)	(0.38)	*p*=0.2648
Cl	103.13± 0.75	100.13 ± 1.56	100.00 ± 0.98	100.05 ± 0.80	100.55 ± 0.95	F=0.6959, df=27 (4,23)
(100-110 mmol/L)	(1.49)	(3.83)	(2.41)	(1.96)	(1.98)	*p*=0.6052

Data are expressed as Mean ± standard error (SE). Standard deviations (SD) are presented in the brackets.

One –way ANOVA followed by Tukey (HSD) post hoc test was performed to seek significant differences between means. Significantly different at $p < 0.05$. ^{C}Differences in respect to control; ^{MgBT}Differences in respect to Mg™.

Significant differences:

Albumin: C *vs* Mg BT: *p*=0.0001; **ALT:** C *vs* Mg Gly: *p*=0.019; **AST:** C *vs* Mg Oxi: *p*=0.0014; C *vs* Mg Gly: *p*=0.0092; **Chol:** C *vs* Mg BT: *p*=0.0074; **Glu:** C *vs* Mg BT: *p*=0.0008; C *vs* Mg Cit: *p*=0.0033; **Prot-T:** C *vs* Mg BT: *p*<0.0001; Mg BT *vs* Mg Cit: *p*=0.0082; **Trig:** C *vs* Mg BT: *p*<0.0001; Mg BT *vs* Mg Cit: *p*=0.0082
