# Supplementary material for: Integrated Systemic and Neurobiological Effects of a Multi-Form Magnesium Supplement Compared to Single Magnesium Forms in Rats
Source: Int J Mol Sci. 2026 Jul 24;27(15):6581. doi: 10.3390/ijms27156581 (PMC13465013; doi:10.3390/ijms27156581)
Supplement: Supplementary file 1 [file ijms-27-06581-s001.zip › Supplementary_Table S5.pdf]

Integrated Systemic and Neurobiological Effects of the Multi-Form Magnesium Supplement Magnesium Breakthrough in Rats Compared to Single Magnesium Forms

Muamer Dizdar [1], Monia Obučić [2], Neira Crnčević [2], Svetlana Dinić [3], Aleksandra Uskoković [3], Jelena Arambašić Jovanović [3], Slavica Borković-Mitić [3], Aleksandra Mladenović [3], Desanka Milanović [3], Smilja Praćer [3], Nataša Nestorović [3], Milica Manojlović-Stojanoski [3], Mirjana Mihailović [3] and Slađan Pavlović [3,*]

[1] Faculty of Science, University of Sarajevo, Zmaja od Bosne 33-35, 71000 Sarajevo, Bosnia and Herzegovina;
muamer.dizdar@pmf.unsa.ba (M.D.)
[2] Faculty of Engineering, Natural and Medical Sciences, International Burch University, Francuske Revolucije bb, 71000 Sarajevo, Bosnia and Herzegovina;
monia.avdic@ibu.edu.ba (M.O.); neira.crncevic@ibu.edu.ba (N.C)
[3] Institute for Biological Research "Siniša Stanković" – National Institute of the Republic of Serbia, University of Belgrade, Bulevar despota Stefana 142, 11108 Belgrade, Serbia;
sdinic@ibiss.bg.ac.rs (S.D.); auskokovic@ibiss.bg.ac.rs (A.U.);
jelena.arambasic@ibiss.bg.ac.rs (J.A.J.); borkos@ibiss.bg.ac.rs (S.B.-M.);
anamikos@ibiss.bg.ac.rs (A.M.); desan@ibiss.bg.asc.rs (D.M.); smilja.pracer@ibiss.bg.ac.rs (S.P.); rnata@ibiss.bg.ac.rs (N.N.) manojlo@ibiss.bg.ac.rs (M.M.-S.);
mirjanam@ibiss.bg.ac.rs (M.M.); sladjan@ibiss.bg.ac.rs (S.Z.P.)
* Correspodence: sladjan@ibiss.bg.ac.rs (S.Z.P.);

Supplementary material

Table S4. ANOVA effects for OGTT.

ANOVA (Control)	DF	F (DFn, DFd)
Treatment (between columns)	4	F (4, 20) = 20.99
Residual (within columns)	20	
Total	24	
**Dunnett's multiple comparisons test**	**Summary**	**Adjusted P Value**
0 vs. 30	****	<0.0001
0 vs. 60	****	<0.0001

ANOVA (Mg BT™)	DF	F (DFn, DFd)
Treatment (between columns)	4	F(4, 25) = 98,13
Residual (within columns)	25	
Total	29	
**Dunnett's multiple comparisons test**	**Summary**	**Adjusted P Value**
0 vs. 30	****	<0.0001
0 vs. 60	****	<0.0001
0 vs. 120	***	0.0002

ANOVA (Mg Oxi)	DF	F (DFn, DFd)
Treatment (between columns)	4	F(4, 25) = 15,52
Residual (within columns)	25	
Total	29	
**Dunnett's multiple comparisons test**	**Summary**	**Adjusted P Value**
0 vs. 30	****	<0.0001
0 vs. 60	****	<0.0001

ANOVA (Mg Cit)	DF	F (DFn, DFd)
Treatment (between columns)	4	F(4, 25) = 46,64
Residual (within columns)	25	
Total	29	
**Dunnett's multiple comparisons test**	**Summary**	**Adjusted P Value**
0 vs. 30	****	<0.0001
0 vs. 60	****	<0.0001
0 vs. 120	**	0.0093

ANOVA (Mg Gly)	DF	F (DFn, DFd)
Treatment (between columns)	4	F(4, 25) = 22,37
Residual (within columns)	25	
Total	29	
**Dunnett's multiple comparisons test**	**Summary**	**Adjusted P Value**
0 vs. 30	****	<0.0001
0 vs. 60	****	<0.0001
0 vs. 120	**	0.0092
