# Supplementary material for: Integrated Systemic and Neurobiological Effects of a Multi-Form Magnesium Supplement Compared to Single Magnesium Forms in Rats
Source: Int J Mol Sci. 2026 Jul 24;27(15):6581. doi: 10.3390/ijms27156581 (PMC13465013; doi:10.3390/ijms27156581)
Supplement: Supplementary file 1 [file ijms-27-06581-s001.zip › Supplementary_Table S6.pdf]

Integrated Systemic and Neurobiological Effects of the Multi-Form Magnesium Supplement Magnesium Breakthrough in Rats Compared to Single Magnesium Forms

Muamer Dizdar [1], Monia Obučić [2], Neira Crnčević [2], Svetlana Dinić [3], Aleksandra Uskoković [3], Jelena Arambašić Jovanović [3], Slavica Borković-Mitić [3], Aleksandra Mladenović [3], Desanka Milanović [3], Smilja Praćer [3], Nataša Nestorović [3], Milica Manojlović-Stojanoski [3], Mirjana Mihailović [3] and Slađan Pavlović [3,*]

[1] Faculty of Science, University of Sarajevo, Zmaja od Bosne 33-35, 71000 Sarajevo, Bosnia and Herzegovina; muamer.dizdar@pmf.unsa.ba (M.D.)

[2] Faculty of Engineering, Natural and Medical Sciences, International Burch University, Francuske Revolucije bb, 71000 Sarajevo, Bosnia and Herzegovina; monia.avdic@ibu.edu.ba (M.O.); neira.crncevic@ibu.edu.ba (N.C)

[3] Institute for Biological Research "Siniša Stanković" – National Institute of the Republic of Serbia, University of Belgrade, Bulevar despota Stefana 142, 11108 Belgrade, Serbia; sdinic@ibiss.bg.ac.rs (S.D.); auskokovic@ibiss.bg.ac.rs (A.U.); jelena.arambasic@ibiss.bg.ac.rs (J.A.J.); borkos@ibiss.bg.ac.rs (S.B.-M.); anamikos@ibiss.bg.ac.rs (A.M.); desan@ibiss.bg.asc.rs (D.M.); smilja.pracer@ibiss.bg.ac.rs (S.P.); rnata@ibiss.bg.ac.rs (N.N.) manojlo@ibiss.bg.ac.rs (M.M.-S.); mirjanam@ibiss.bg.ac.rs (M.M.); sladjan@ibiss.bg.ac.rs (S.Z.P.)

* Correspodence: sladjan@ibiss.bg.ac.rs (S.Z.P.);

Supplementary material

Table S6. Chemical composition of the rat food IG–Z–00117. Complete mixture. GEBI d.o.o., Čantavir, Serbia (e-mail: office@gebi.rs; www.gebi.rs)

**Chemical composition of rat diet**					
**General**		**Vitamins**		**Minerals**	
Proteins	min 20.00%	Vit A	min 12.000 U/kg	Zinc	min 30 mg/kg
Fat	min 5.00%	Vit D3	min 2.000 U/kg	Copper	min 15.00 mg/kg
Humidity	max 13.5%	Vit E	min 30.00 mg/kg	Iron	min 60 mg/kg
Cellulose	max 5.00%	Vit B1	min 4.00 mg/kg	Manganese	min 60 mg/kg
Ashes	max 8.00%	Vit B2	min 7.00 mg/kg	Iodine	min 0.15 mg/kg
Calcium	min 0.95%	Vit B6	min 6 mg/kg	Selenium	min 0.10 mg/kg
Phosphorus	min 0.70%	Vit B12	min 0.05 mg/kg		
Lysine	min 0.60%	Folic acid	min 1.0 mg/kg	**Antioxidants**	
Methionine	min 0.60%	Choline chloride	1.000.00 mg/kg	E-321, E-320, E-338, E202	min 125 mg/kg
Tryptophan	max 0.15%	Niacin	20.00 mg/kg		
		Pantothenic acid	min 10.00 mg/kg		
